# Lysine methyltransferase methyltransferase-like 13 regulates bone marrow mesenchymal stem cells osteo-adipogenic differentiation and senescence in osteoporosis via the Foxa1/HES-1 axis

**DOI:** 10.1093/stcltm/szag049

**Published:** 2026-07-28

**Authors:** Ying Liu, Ao Wang, Ziwen Liu, Zhengwei Qin, Lei Yin, Xinyue Wang, Mingyu He, Tao Li, Yanquan Wang, Longhao Chen, Zhuo Liu, Jiayi Liu, Binsha Gu, Shichao Yang, Hui Liang, Yang Li, Xiaofei Zheng, Shuixing Zhang, Baofeng Yang, Ye Yuan, Lei Yang

**Affiliations:** Department of Pharmacology (State Key Laboratory of Frigid Zone Cardiovascular Diseases (SKLFZCD), State-Province Key Laboratories of Biomedicine Pharmaceutics of China, Key Laboratory of Cardiovascular Research, Ministry of Education), College of Pharmacy, Harbin Medical University, Harbin 150081, China; Department of Pharmacology (State Key Laboratory of Frigid Zone Cardiovascular Diseases (SKLFZCD), State-Province Key Laboratories of Biomedicine Pharmaceutics of China, Key Laboratory of Cardiovascular Research, Ministry of Education), College of Pharmacy, Harbin Medical University, Harbin 150081, China; Department of Orthopedic Surgery, The Second Affiliated Hospital of Harbin Medical University, Harbin 150081, China; Department of Pharmacy at the Second Affiliated Hospital, State Key Laboratory of Frigid Zone Cardiovascular Diseases (SKLFZCD), Harbin Medical University, Harbin 150081, China; Department of Clinical Pharmacology, College of Pharmacy, Harbin Medical University, Harbin 150081, China; Department of Pharmacy at the Second Affiliated Hospital, State Key Laboratory of Frigid Zone Cardiovascular Diseases (SKLFZCD), Harbin Medical University, Harbin 150081, China; Department of Clinical Pharmacology, College of Pharmacy, Harbin Medical University, Harbin 150081, China; Department of Pharmacology (State Key Laboratory of Frigid Zone Cardiovascular Diseases (SKLFZCD), State-Province Key Laboratories of Biomedicine Pharmaceutics of China, Key Laboratory of Cardiovascular Research, Ministry of Education), College of Pharmacy, Harbin Medical University, Harbin 150081, China; Department of Pharmacology (State Key Laboratory of Frigid Zone Cardiovascular Diseases (SKLFZCD), State-Province Key Laboratories of Biomedicine Pharmaceutics of China, Key Laboratory of Cardiovascular Research, Ministry of Education), College of Pharmacy, Harbin Medical University, Harbin 150081, China; Department of Pharmacology (State Key Laboratory of Frigid Zone Cardiovascular Diseases (SKLFZCD), State-Province Key Laboratories of Biomedicine Pharmaceutics of China, Key Laboratory of Cardiovascular Research, Ministry of Education), College of Pharmacy, Harbin Medical University, Harbin 150081, China; Department of Pharmacology (State Key Laboratory of Frigid Zone Cardiovascular Diseases (SKLFZCD), State-Province Key Laboratories of Biomedicine Pharmaceutics of China, Key Laboratory of Cardiovascular Research, Ministry of Education), College of Pharmacy, Harbin Medical University, Harbin 150081, China; Department of Orthopedics at the First Affiliated Hospital, State Key Laboratory of Frigid Zone Cardiovascular Diseases (SKLFZCD), Harbin Medical University, Harbin 150081, China; Department of Pharmacology (State Key Laboratory of Frigid Zone Cardiovascular Diseases (SKLFZCD), State-Province Key Laboratories of Biomedicine Pharmaceutics of China, Key Laboratory of Cardiovascular Research, Ministry of Education), College of Pharmacy, Harbin Medical University, Harbin 150081, China; Department of Pharmacology (State Key Laboratory of Frigid Zone Cardiovascular Diseases (SKLFZCD), State-Province Key Laboratories of Biomedicine Pharmaceutics of China, Key Laboratory of Cardiovascular Research, Ministry of Education), College of Pharmacy, Harbin Medical University, Harbin 150081, China; Department of Pharmacy at the Second Affiliated Hospital, State Key Laboratory of Frigid Zone Cardiovascular Diseases (SKLFZCD), Harbin Medical University, Harbin 150081, China; Department of Clinical Pharmacology, College of Pharmacy, Harbin Medical University, Harbin 150081, China; Department of Pharmacy at the Second Affiliated Hospital, State Key Laboratory of Frigid Zone Cardiovascular Diseases (SKLFZCD), Harbin Medical University, Harbin 150081, China; Department of Clinical Pharmacology, College of Pharmacy, Harbin Medical University, Harbin 150081, China; Department of Pharmacy at the Second Affiliated Hospital, State Key Laboratory of Frigid Zone Cardiovascular Diseases (SKLFZCD), Harbin Medical University, Harbin 150081, China; Department of Clinical Pharmacology, College of Pharmacy, Harbin Medical University, Harbin 150081, China; Department of Pharmacology (State Key Laboratory of Frigid Zone Cardiovascular Diseases (SKLFZCD), State-Province Key Laboratories of Biomedicine Pharmaceutics of China, Key Laboratory of Cardiovascular Research, Ministry of Education), College of Pharmacy, Harbin Medical University, Harbin 150081, China; Center for Endemic Disease Control, Chinese Center for Disease Control and Prevention, Harbin Medical University, Harbin 150081, China; NHC Key Laboratory of Etiology and Epidemiology, (Harbin Medical University), Joint Key Laboratory of Endemic Diseases (Harbin Medical University; Guizhou Medical University; Xi’an Jiaotong University), Heilongjiang Provincial Key Laboratory of Trace Elements and Human Health; Key Laboratory of Etiology and Epidemiology, Education Bureau of Heilongjiang Province, Harbin Medical University, Harbin 150081, China; Department of Sports Medicine, The First Affiliated Hospital, Guangdong Provincial Key Laboratory of Speed Capability, The Guangzhou Key Laboratory of Precision Orthopedics and Regenerative Medicine, School of Medicine, Jinan University, Guangzhou, 510630, China; Department of Radiology, The First Affiliated Hospital of Jinan University, Guangzhou, 510627, China; Department of Pharmacology (State Key Laboratory of Frigid Zone Cardiovascular Diseases (SKLFZCD), State-Province Key Laboratories of Biomedicine Pharmaceutics of China, Key Laboratory of Cardiovascular Research, Ministry of Education), College of Pharmacy, Harbin Medical University, Harbin 150081, China; Department of Pharmacy at the Second Affiliated Hospital, State Key Laboratory of Frigid Zone Cardiovascular Diseases (SKLFZCD), Harbin Medical University, Harbin 150081, China; Department of Orthopedics at the First Affiliated Hospital, State Key Laboratory of Frigid Zone Cardiovascular Diseases (SKLFZCD), Harbin Medical University, Harbin 150081, China; Key Laboratory of Hepatosplenic Surgery of Ministry of Education, The First Affiliated Hospital of Harbin Medical University, Harbin 150081, China; NHC Key Laboratory of Cell Transplantation, The First Affiliated Hospital of Harbin Medical University, Harbin 150081, China

**Keywords:** osteoporosis, METTL13, BMSCs, osteo-adipogenic differentiation, organ aging

## Abstract

**Purpose:**

Recent research indicates that the senescence of bone marrow mesenchymal stem cells (BMSCs) disrupts the osteo-adipogenic balance, a primary factor contributing to the development of osteoporosis. Our previous findings have implicated methyltransferases in this process, among which methyltransferase-like 13 (METTL13) has been established to regulate cell fate, although its role in osteoporosis has yet to be determined.

**Methods:**

Bone formation was assessed using micro-computed tomography and hematoxylin and eosin staining. Protein expression in bone tissues was examined immunohistochemically, and cellular mRNA and protein levels were determined using quantitative reverse transcription-polymerase chain reaction (qRT-PCR) and western blotting. Cellular senescence was evaluated based on **β**-galactosidase staining, and osteogenic and adipogenic differentiation was examined using alkaline phosphatase, Alizarin Red S, and Oil Red O staining. Protein interactions and DNA binding were determined using co-immunoprecipitation and chromatin immunoprecipitation.

**Results:**

METTL13 expression was significantly enhanced in ovariectomy-induced senescent bone and BMSCs, whereas METTL13 knockdown markedly reversed etoposide-induced cellular senescence. By binding to forkhead box protein A1 (Foxa1), METTL13 promotes the preferential differentiation of BMSCs into adipocytes, as opposed to osteocytes. Moreover, Foxa1 had effects opposite to those of METTL13 on BMSC differentiation, inhibiting the nuclear entry of METTL13. Notably, blocking the nuclear import of Foxa1 suppressed the transcriptional expression of HES-1, which promoted the adipogenic differentiation of BMSCs and inhibited osteogenic differentiation.

**Conclusions:**

Our findings in this study revealed the mechanisms whereby METTL13 promotes BMSC senescence and disrupts BMSC differentiation, on the basis of which, we identified the METTL13–Foxa1–HES-1 axis as a potential therapeutic target for treatment of osteoporosis.

## Graphical Abstract

**Graphical Abstract:**
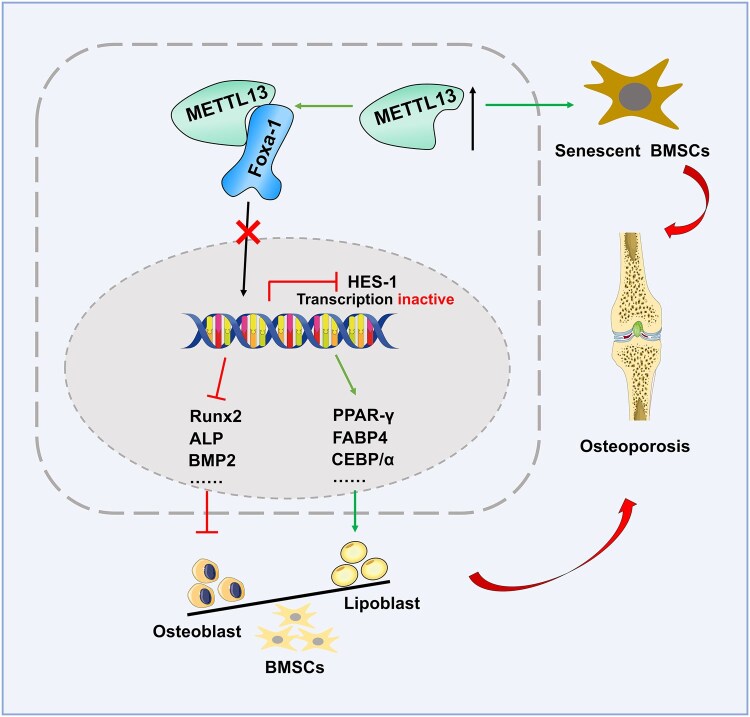

Significance statement In this study, we identified methyltransferase-like 13 (METTL13) as a factor that promotes bone marrow stem cell aging and adipocyte formation, thereby contributing to the development of osteoporosis. Mechanistically, METTL13 binds to forkhead box protein A1 (Foxa1), blocking its nuclear entry and reducing the expression of HES-1. These findings highlight the potential utility of METTL13 and the Foxa1/HES-1 axis as diagnostic markers and therapeutic targets in osteoporosis.

## Introduction

With global population aging, osteoporosis has become the most common metabolic bone disease compromising human health and safety.^1^ Osteoporosis is characterized by imbalanced bone metabolism, increased resorption, reduced formation, and disrupted homeostasis.^2^ Bone marrow mesenchymal stem cells (BMSCs) are common progenitor cells from which osteoblasts and adipocytes are derived. The balance between osteogenic and adipogenic differentiation is regulated by factors maintaining bone homeostasis.^3^ During aging or in response to certain pathological stimuli (such as hormonal imbalance), BMSCs show increased differentiation into adipocytes, resulting in an imbalance between bone mass and fat, ultimately leading to bone loss.^4^ The differentiation and self-renewal of BMSCs are regulated by the combined effects of the extracellular matrix, oxidative stress, mechanical stress, epigenetic modification, and intracellular signaling factors, which are mediated by the expression of key adipogenic and osteogenic genes.^5–9^

Given the genetic and phenotypic heterogeneity of aging, it is a complex pathological process with manifestations that vary across biological systems, tissues, and functions.^10^^,^^11^ In this context, the findings of recent studies have shown that senescent cells play a causal role in BMSC differentiation and bone formation.^12–14^ Cell senescence is associated with dysfunctional osteogenic differentiation and impairs the regenerative potential of BMSCs.^13–15^ For example, nucleosome assembly protein 1-like 2 (NAP1L2), a histone chaperone, induces cell senescence and recruits SIRT1 to deacetylate H3K14ac, thereby upregulating osteogenic gene expression and suppressing the osteogenic differentiation of BMSCs. Moreover, it has been found that these effects can be alleviated by the anti-aging reagent nicotinamide mononucleotide.^14^ However, the specific molecular mechanisms whereby age-mediated stem cell differentiation contributes to the progression of osteoporosis remain to be determined.

Methyltransferases comprise an important group of widely expressed enzymes that play essential roles in the synthesis or degradation of physiologically active substances, as well as in gene expression, thereby contributing to growth and development by catalyzing the methylation of DNA, RNA, and proteins.^16^^,^^17^ Our research group has been conducting long-term studies on the function of methyltransferase-related genes in the diagnosis and treatment of osteoporosis,^8^^,^^18^ and we have demonstrated that by separately targeting pre-miR-320 methylation and autophagic production, the m6A methyltransferases METTL3 and METTL14 function as osteoporosis suppressor genes that promote the osteogenic differentiation of BMSCs.^8^^,^^18^ Among other members of this family, methyltransferase-like 13 (METTL13), which has dual methyltransferase properties, has been reported to catalyze the dimethylation of eukaryotic elongation factor 1A (eEF1A) at lysine 55 (eEF1AK55me2), thereby promoting protein synthesis in RAS-driven cancers.^19^^,^^20^ METTL13 is widely expressed in human cells and tissues, and plays roles in determining cellular fate and disease development.^21^^,^^22^ Recently, researchers have confirmed that HN1L positively regulates the expression of METTL13 at the transcriptional level, thereby promoting the development of hepatocellular carcinoma by upregulating the expression of TCF3 and ZEB1.^23^ In addition, it has been demonstrated that by targeting METTL13, which functions as a potential inhibitor of cell apoptosis, miR-16 promotes the apoptosis of tumor cells.^24^ However, the role of METTL13 in the development of osteoporosis remains to be established.

In this study, we demonstrated that by promoting senescence and regulating the osteo-adipogenic balance in BMSCs, METTL13 plays a key role in the progression of osteoporosis. Mechanistically, our findings revealed that the interaction between METTL13 and the transcription factor forkhead box protein A1 (Foxa1) suppresses HES-1 transcription, thereby inhibiting the osteogenic differentiation of BMSCs in favor of adipogenesis, and thus contributing to the development of osteoporosis. On the basis of these findings, we identified functional associations among METTL13, Foxa1, and HES-1 in osteoporosis, thereby implying that the METTL13–Foxa1–HES-1 axis could serve as a promising therapeutic target for the treatment of this pernicious bone disease.

## Methods

### Cell culturing and treatment

Mouse BMSCs (MUBMX-01001: C57BL/6; Cyagen) were cultured in complete medium (MUBMX-90011; Cyagen) at 37 °C in a 5% CO_2_ atmosphere. Cells at passages 3–5 were confirmed by flow cytometry to be CD29+, CD90+, CD105+, and CD34−/CD45− and used for experiments. Upon reaching 80%-90% confluence, the cells were detached using 0.25% trypsin-EDTA and seeded at 2 × 10^4^ cells/cm^2^. HEK-293T cells (FH0244; FuHeng Biology) were cultured in DMEM (Sigma) supplemented with 10% serum and 1% penicillin–streptomycin under identical conditions. For primary BMSCs, bone marrow flushed from mouse femurs and tibias was filtered, centrifuged, and resuspended in culture medium. Senescence was induced by treating BMSCs with 5 μM etoposide (MCE; 33419-42-0) for 48 h.

### Human bone samples

Bone specimens were obtained from the Department of Orthopedics, First Affiliated Hospital of Harbin Medical University. Healthy controls were collected from trauma patients under 40 years, whereas osteoporotic samples were obtained from patients over 65 with severe osteoporosis. This study was approved by the Experimental Animal Ethics Committee of Harbin Medical University.

### Construction of METTL13 knockdown or overexpressing mouse models

Knockdown and overexpression of METTL13 in mice were achieved via intratibial injection of adeno-associated virus 9 (AAV9). Six-week-old female C57BL/6 mice received 10 μL (1 × 10^10^ PFU/mL) of AAV9-shMETTL13 (knockdown) or AAV9-METTL13 (overexpression), along with the corresponding negative controls (Hanbio, Genechem, China). Infection efficiency was assessed by quantitative reverse transcription-polymerase chain reaction (qRT-PCR) and western blot analyses on day 7 post-injection.

### Mouse osteoporosis model

Eight-week-old female C57BL/6 mice underwent bilateral ovariectomy or sham surgery 1 week post-injection. Ovaries were removed via a dorsal incision, and femurs were collected at 8 weeks post-surgery.

### Bone micro-computed tomography (**μ**CT)

Having removed attached muscle tissues, femurs were cleaned, fixed in 4% paraformaldehyde at 4 °C for 48 h, and decalcified in 10% EDTA for 3 weeks. Bone morphology and micro-structure were analyzed using a Skyscan1076 micro-CT scanner (SkyScan, Belgium). To minimize variability, all image segmentations and analyses were performed by a single blinded operator.

### Western blot analysis

Proteins were extracted from cells and ground bone tissues via lysis overnight at 4 °C using RIPA buffer (Beyotime, China). Following centrifugation at 13 500 rpm, protein concentrations were determined using a BCA kit (Beyotime, China). Samples (30 µg) were separated using SDS-PAGE, transferred onto nitrocellulose membranes, and blocked with 5% skimmed milk for 1 h. Thereafter, the membranes were incubated with primary antibodies overnight at 4 °C (see Table S2), followed by incubation with secondary antibodies (anti-rabbit or anti-mouse, 1:5000; ABCAM) for 1 h at room temperature. Bands were visualized using Odyssey CLX and quantified using Li-COR software.

### Nuclear–cytosolic fractionation

Nuclear and cytosolic fractions were isolated from 1 × 10^6^ cells using an NE-PER kit (78833; Thermo Fisher Scientific). The cells were sequentially lysed with cytoplasmic reagents I and II, and the supernatant (cytoplasmic fraction) was collected after centrifugation at 16 000 × *g* for 5 min. The remaining pellet was resuspended in nuclear extraction reagent, incubated on ice for 40 min with vortexing at 10-min intervals, and centrifuged at 16 000 × *g* for 10 min to obtain the nuclear fraction.

### Immunohistochemical staining

Bone tissues were decalcified in 10% EDTA for 28 days, dehydrated using an alcohol and xylene gradient for 20 h, and embedded in paraffin. Sections (4 µm) were de-paraffinized, subjected to antigen retrieval (AR0022; BOSTER) for 30 min at 4 °C, and incubated overnight with primary antibodies. Following incubation with secondary antibodies (PV-6001; ORIGENE) for 20 min at room temperature, the sections were developed using DAB (ZLI-9018; ORIGENE) for 5 min and counterstained with hematoxylin (BL702B; Biosharp) for 2 min. Subsequent to dehydration, the sections were mounted with neutral balsam (G8590; Solarbio). As primary antibodies, we used rabbit anti-METTL13 (1:1000: ab186002; ABCAM) and rabbit anti-Foxa1 (1:1000: GTX100308; GeneTex).

### Senescence-associated **β**-galactosidase staining

β-galactosidase staining was performed on BMSCs using a commercial kit (C0605; Beyotime). Cells were treated with 5 µM etoposide for 48 h or transfected with siMETTL13 for 24 h. Following fixation, the cells were incubated with staining solution overnight at 37 °C.

### Co-immunoprecipitation (Co-IP)

Tissues were lysed in RIPA buffer (Beyotime, China), sonicated, and centrifuged. The resulting supernatants were incubated with magnetic beads overnight at 4 °C, washed three times with PBST, and then incubated with antibodies overnight at 4 °C. Bound proteins and 10% whole-cell lysates were analyzed via immunoblotting. As antibodies, we used rabbit/mouse anti-HA (T0050; AFFINITY and AE008; Abclonal), mouse/rabbit anti-FLAG (66008-3-IG; Proteintech and AE004; Abclonal), and mouse anti-IgG (AC011; Abclonal).

### Hematoxylin and eosin staining

Mouse femurs were cleaned, fixed in 4% paraformaldehyde for 24 h, decalcified in 10% EDTA for 28 days, and dehydrated through an ethanol and xylene gradient for 20 h. After paraffin embedding, 4-µm sections were de-waxed and stained with hematoxylin and eosin (H&E: G1120; Solarbio). Following dehydration, the sections were mounted with neutral balsam (G8590; Solarbio). Images obtained using a confocal microscope were analyzed using LAS V4.13 software. Bone growth was assessed based on the trabecular bone density at the femoral head.

### RNA extraction

Total RNA was extracted from BMSCs using TRIzol reagent (Invitrogen). Cells were lysed at room temperature, mixed with 200 µL of chloroform, vigorously shaken, left to stand for 3 min, and centrifuged at 13 500 rpm for 15 min at 4 °C. The aqueous phase was transferred to fresh tubes, mixed with 500 µL of isopropanol, incubated for 30 min, and centrifuged at 13 500 rpm for 10 min at 4 °C. The resulting RNA pellets were washed with 1 mL of 70% ethanol, centrifuged at 10 600 rpm for 5 min, air-dried, and dissolved in 10 µL of DEPC-treated water (ZS105-2; ZOMANBIO).

### qRT-PCR

RNA expression was measured by qRT-PCR. Complementary DNA was synthesized using a commercial transcription kit (00676299; Thermo Fisher Scientific), with samples being amplified using a 7500HT rapid real-time PCR system (Applied Biosystems) in conjunction with a SYBR Green PCR master mix (31598800; Roche). mRNA expression was normalized to that of GAPDH. The sequences of the primers used for amplification are listed in Table S1.

### Alizarin Red S (ARS) staining

To visualize calcium deposits, cells were fixed with 4% formaldehyde for 15 min at 37 °C, stained with ARS solution (S0141; Cyagen) for 5 min, and rinsed with phosphate-buffered saline (PBS). Stained cells were imaged under a Nikon Eclipse TS100 light microscope (Nikon) and quantified using Image-Pro Plus software.

### Oil red O staining

To evaluate lipid accumulation, we performed Oil Red O staining. Cells were fixed with 4% formaldehyde for 15 min at 37 °C, washed with PBS, and stained with Oil Red O staining solution (T171123G001; Cyagen Biosciences) for 15 min. The stained cells were imaged under a Nikon Eclipse TS100 light microscope and quantified using Image-Pro Plus software.

### Alkaline phosphatase (ALP) assay

To assess osteoblast activity, we performed staining using ALP. Cells were fixed with 4% formaldehyde for 15 min at 37 °C, washed with deionized water, and then incubated for 2 min at room temperature in a staining solution (SLBS7218; Sigma-Aldrich). Having thereafter added naphthol AS-BI Alkaline solution (SLBQ6470; Sigma-Aldrich), the cells were incubated in the dark for 15 min, followed by counterstaining with hematoxylin (SLBQ6779; Sigma-Aldrich) for 5 min and subsequent rinsing. Images were obtained using a Nikon Eclipse TS100 light microscope and analyzed using ImageJ software (NIH, USA).

### Plasmid and siRNA transfection

BMSCs were seeded in six-well plates at 1 × 10^5^ cells/mL and transfected with plasmids encoding METTL13, Foxa1, or negative control using Lipofectamine 3000 (MAN0009872; Invitrogen). Similarly, cells were transfected with siRNAs or control oligonucleotides using Lipofectamine RNAiMAX (MAN0007825; Invitrogen). Subsequent assays were performed at 24 h post-transfection. The siRNA sequences used for transfection are listed in Table S3.

### Chromatin immunoprecipitation (ChIP)

ChIP was performed using a commercial kit (17-371; Sigma). Cells were cross-linked with formaldehyde and DNA was sheared by sonication. Protein–DNA complexes were immunoprecipitated using target antibodies. Following elution, cross-linking was reversed by overnight incubation in NaCl. DNA was purified using spin columns and analyzed by qRT-PCR.

### Statistical analysis

Data were analyzed using Prism 7 software, with values expressed as the means ± SEM. Statistical analyses were performed using Student’s *t*-test (two-tailed) for two-group comparisons. All experiments were independently repeated at least three times. *P*-values < 0.05 were considered statistically significant.

## Results

### METTL13 accelerates the development of osteoporosis in vivo

**Figure 1.**
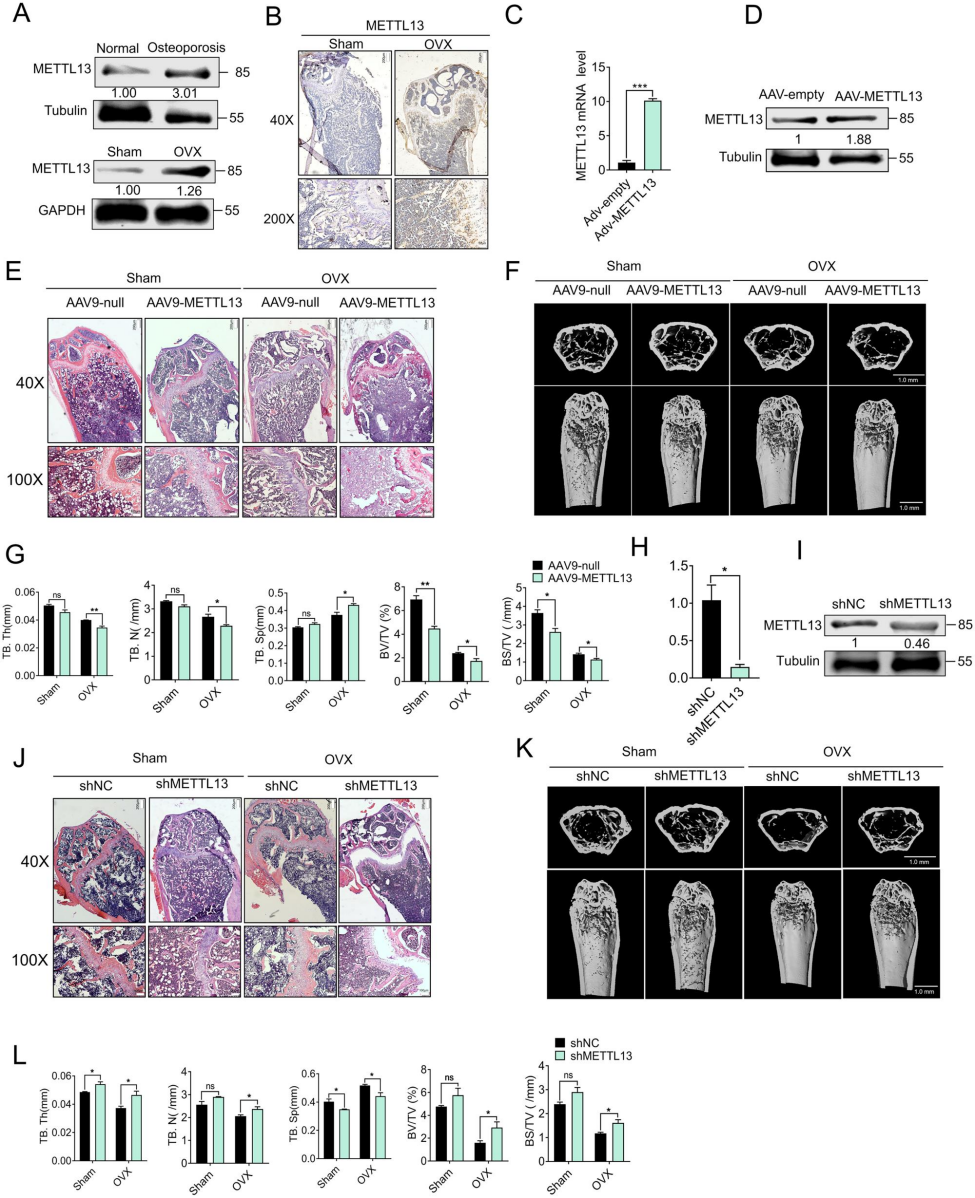
Methyltransferase-like 13 (METTL13) accelerates the development of osteoporosis *in vivo*. (A) Protein expression of METTL13 in human bone from normal and osteoporosis and in sham-operated (Sham) and ovariectomized (OVX) mice. (B) Representative immunohistochemistry (IHC) images of METTL13 expression in the bone of sham and OVX mice. Scale bars: 200 μm (upper); 50 μm (bottom). (C and D) quantitative reverse transcription-polymerase chain reaction (qRT-PCR) (C) and western blot (D) detected the efficiency of adeno-associated virus type 9 (AAV9) of METTL13 overexpression in mice bone tissue. (E) Representative hematoxylin and eosin (H&E) staining images of the AAV9-null or AAV9-METTL13 mouse femur. Scale bar: 200 μm (upper) and 10 μm (lower). *n* = 3. (F) Representative μCT images of trabecular bone of the femoral metaphysis (top) (Continued)and entire proximal femur (bottom) showing the exacerbating effect of AAV9-METTL13 on osteoporosis development in OVX mice. Scale bars, 1.0 mm. (G) μCT analysis showing the change of trabecular thickness (Tb. Th), trabecular number (Tb. N), trabecular separation (Tb. Sp), bone surface area tissue volume ratio (BS/TV) and bone-volume/tissue-volume ratio (BV/TV). *n* = 4. (H and I) qRT-PCR (H) and western blot (I) detected the efficiency of AAV9 of METTL13 knockdown in mice bone tissue. (J) Representative H&E staining images of the AAV9-shNC or AAV9-shMETTL13 mouse femurs. Scale bar: 200 μm (upper) and 10 μm (lower). *n* = 3. (K) Representative μCT images of trabecular bone of the femoral metaphysis (top) and entire proximal femur (bottom) showing the rescuing effects of METTL13 silencing (AAV9-shMETTL13) on the OVX mice. Scale bars: 1.0 mm. (L) μCT analysis showing the change of Tb.Th, Tb. N, Tb.Sp, BV/TV and BS/TV. *n* = 4. Scale bar: 400 μm. *n* = 3. Data are expressed as mean ± SEM. ns: no significance; *P < 0.05; **P < 0.01; ***P < 0.001.

To determine the role of METTL13 in osteoporosis, we examined its expression in bone tissues obtained from patients with osteoporosis and ovariectomized (OVX) model mice. Compared with normal controls, levels of METTL13 protein were markedly higher in osteoporotic bones derived from humans and mice (Figure 1A), with immunohistochemical analysis further confirming elevated METTL13 expression in the bone tissues of OVX mice relative to those detected in sham-operated mice (Figure 1B).

To determine whether METTL13 regulates bone formation, we performed gain-of-function studies by injecting AAV9 carrying the METTL13 gene into the tibia of mice 7 days prior to OVX surgery. qRT-PCR and western blot analyses confirmed an overexpression of METTL13 in bone tissues (Figure 1C, D). Two months post-surgery, H&E staining revealed that METTL13 overexpression significantly reduced femoral trabecular number (Tb.N) and trabecular formation (Figure 1E).

μCT analysis further showed that METTL13 overexpression inhibited bone formation, as evidenced by reduced bone volume fraction (BV/TV) and bone surface area to tissue volume ratio (BS/TV) in sham-operated mice. Moreover, in both sham-operated and OVX mice, overexpression of METTL13 exacerbated the impairment of proximal tibia development attributable to OVX, leading to further reductions in BV/TV, BS/TV, Tb.N, and trabecular thickness (Tb.Th), along with increases in trabecular separation (Tb. Sp) (Figure 1F, G).

To confirm these findings, we knocked down METTL13 in vivo using AAV9 shMETTL13, thereby reducing METTL13 mRNA and protein levels by approximately 80% and 50%, respectively, compared with those in mice treated with the control shRNA (Figure 1H, I). Subsequently, we performed H&E staining and μCT to assess the effects on bone formation 2 months after OVX surgery. Both the shMETTL13-treated sham-operated and OVX mice were characterized by marked enhancements in Tb.N (Figure 1J). In addition, μCT analysis of cortical and trabecular bone in the proximal tibia revealed increases in Tb.Th and reductions in Tb.Sp in the sham-operated mice with METTL13 knockdown. Moreover, compared with the shNC mice, we obtained evidence of a considerably higher bone mass in the shMETTL13-treated OVX mice, as indicated by increases in Tb.Th, Tb.N, BS/TV, and BV/TV and a reduction in Tb.Sp (Figure 1K, L). Collectively, these findings indicate that METTL13 inhibits bone formation and promotes the progression of osteoporosis in vivo.

### METTL13 is positively correlated with bone aging and BMSC senescence

**Figure 2.**
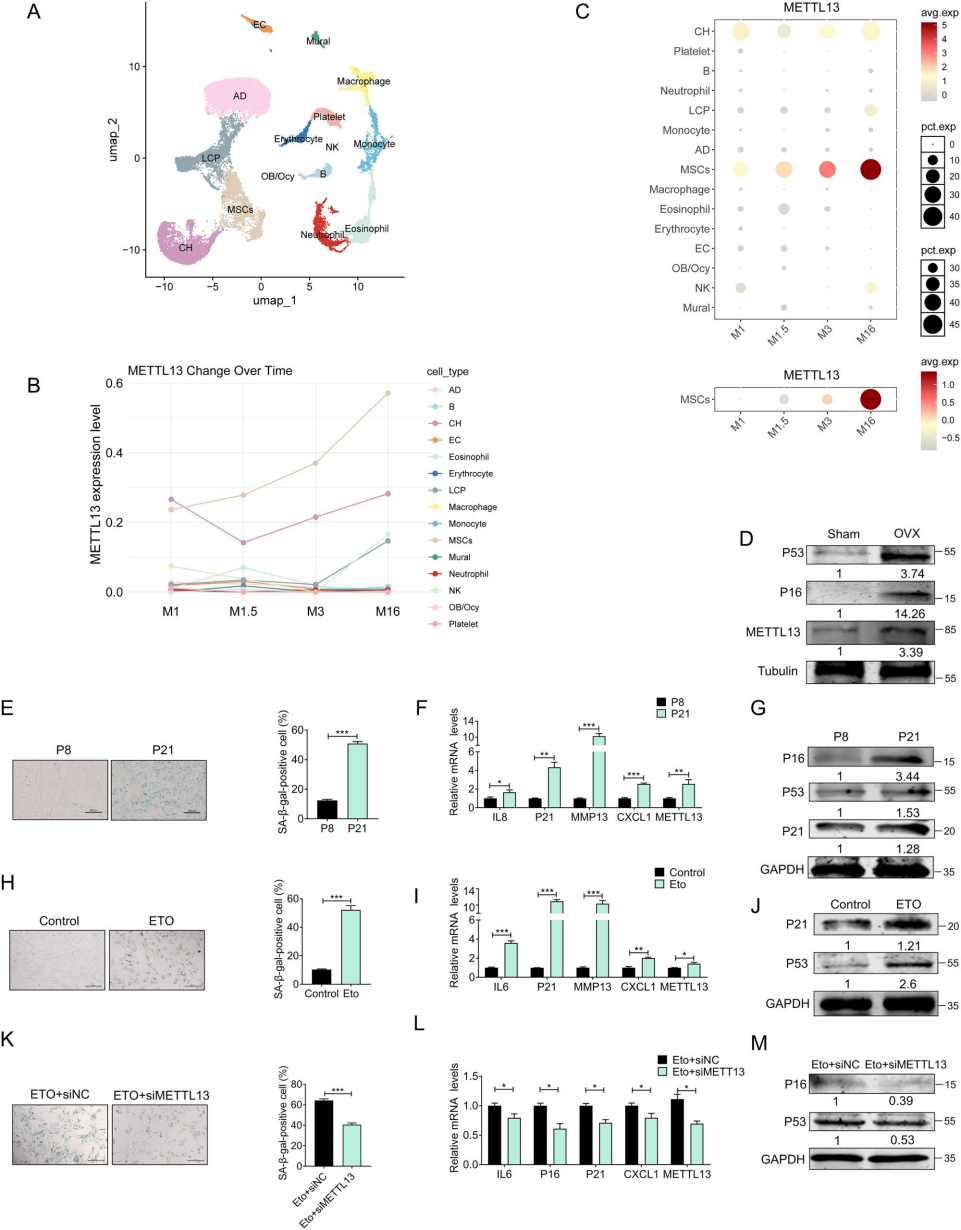
Methyltransferase-like 13 (METTL13) is positively collected with bone aging and bone marrow mesenchymal stem cells (BMSCs) senescence. (A) The t-SNE plot of cells in GSE1 GSE145477. (B) The expression of the METTL13 gene changes over time in different cells. (G) Dot plots of the METTL13 gene expressions in different cells. (D)The protein expression levels of P53, P16, and METTL13 in the bone tissue from the sham-operated (Sham) and the ovariectomized (OVX) mice. (E) Representative images of passage 8 (P8) and passage 21 (P21) SA-β-gal-positive BMSCs (Continued)(blue). Scale bar: 200 μm. (F) The mRNA expression levels of age-related genes (IL8, P21, MMP13, CXCL1, and METTL13) in passage 8 (P8) and passage 21 (P21) BMSCs. (G) Western blot results for protein expression levels of P16, P53, and P21 in passage 8 (P8) and passage 21 (P21) BMSCs. (H) Beta-galactosidase staining in BMSCs with or without 5 μM etoposide treatment for 48 h. SA-β-gal-positive BMSCs are blue. Scale bar: 200 μm. (F, G) Relative mRNA (I) and protein (J) expression levels of senescence-related genes (IL6, P21, MMP13, CXCL1, METTL13, and P53) in BMSCs with or without 5 μM etoposide treatment for 48 h were detected by reverse transcription-polymerase chain reaction (RT-PCR) and western blot. (L) The knockdown efficiency of METTL13 was detected by RT-PCR. (K-M) Identification of the reverse effect of METTL13 knockdown on BMSCs treated with 5 μM etoposide for 48 h by β-galactosidase staining (K), RT-PCR (L) and western blot (M). Data are expressed as mean ± SEM. *P < 0.05; **P < 0.01; ***P < 0.001.

Stem cells senescence has been shown to be closely associated with the pathogenesis of multiple aging-related diseases, including osteoporosis.^13^^,^^14^ To determine whether METTL13 regulates osteoporosis via stem cell senescence, we analyzed single-cell RNA sequencing data obtained for the bones of mice at 1, 1.5, 3, and 16 months of age (GEO: GSE145477) (Figure 2A). With increasing age, we observed a progressive increase in METTL13 expression in mesenchymal stem cells (Figure 2B, C). On the basis of findings regarding METTL13 expression, the aging phenotype, and osteoporosis in mice, we hypothesized that METTL13 may regulate osteoporosis via the aging of BMSCs. Consistent with this assumption, compared with the sham-operated mice, bone tissues from OVX mice were characterized by significantly elevated expression of the senescence markers p16 and p53, along with increases in METTL13 protein levels, thereby providing evidence of a positive association between METTL13 and bone aging (Figure 2D). To further examine this association, we established a cellular senescence model using BMSCs, in which senescence was induced by continuously culturing early-passage cells until passage 21. Compared with that in the passage 8 cells, senescence-associated β-galactosidase staining confirmed a substantial increase in the percentage of senescent cells among those that had undergone 21 passages (Figure 2E). Moreover, we found that in senescent BMSCs, METTL13 expression was notably upregulated, coinciding with increases in the expression of age-related genes (Figure 2F, G). On the basis of previous findings indicating that the topoisomerase inhibitor etoposide can induce premature cellular senescence via DNA damage,^14^ we treated BMSCs with etoposide and similarly detected significant increases in BMSC senescence, as evidenced by increases in the percentage of β-gal-positive cells and upregulation of age-related gene expression (Figure 2H-J). Importantly, following knockdown of METTL13 in BMSCs (Figure 2K), we found that a loss of METTL13 function was associated with a significant retardation of etoposide-induced senescence (Figure 2K-M). Collectively, these findings revealed that METTL13 promotes the progression of bone aging and senescence of BMSCs.

### METTL13 intrinsically controls the fate of BMSCs

Given that osteoporosis results from an imbalance between the osteogenic and adipogenic differentiation of BMSCs, we hypothesized that METTL13 influences BMSC adipogenesis. To verify this conjecture, BMSCs isolated from control and METTL13-overexpressing mice were induced towards osteogenic or adipogenic lineages. ARS staining revealed fewer calcium deposits in the METTL13-overexpressing mice after 7 days of osteogenic induction (Figure S1A). Conversely, Oil Red O staining indicated an increase in lipid droplet formation after 8 days of adipogenic induction (Figure S1B). Thus experimentally inducing the expression of METTL13 in vivo can contribute to determining the fate of BMSCs.

**Figure 3.**
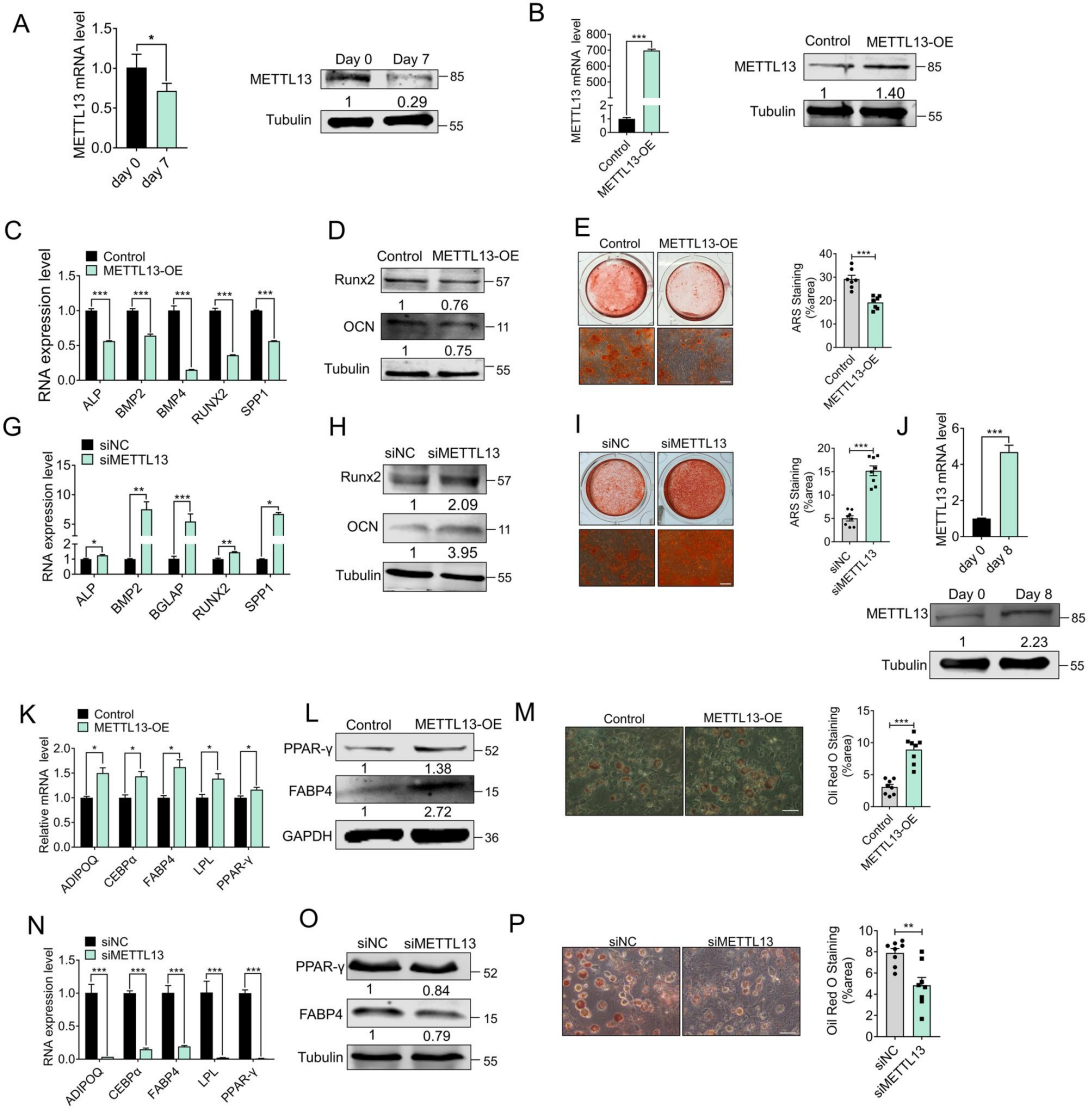
Methyltransferase-like 13 (METTL13) intrinsically controls bone marrow mesenchymal stem cells (BMSCs) fate decisions. (A) Changes of METTL13 mRNA and protein levels after 7 days of osteogenic induction in BMSCs. (B) Transfection efficiency of METTL13 overexpression detected by quantitative reverse transcription-polymerase chain reaction (qRT-PCR) and western blot. (C) mRNA expression levels of osteogenesis-related genes (ALP, BMP2, BGLAP, RUNX2, and SPP1) after 7 days of osteogenic induction with or without METTL13 overexpression. *n* = 3. (D) Western blot confirmed the protein expression change of RUNX2 and osteocalcin (OCN) with or without METTL13 overexpression. (E) Calcium nodule formation ability of BMSCs after METTL13 overexpression detected by Alizarin Red S (ARS) staining. Scale bar upper: 2 μm; lower: 200 μm. *n* = 7. (F) Changes of mRNA expression levels of osteogenesis-related genes (alkaline phosphatase [ALP], bone morphogenetic protein 2 [BMP2], bone gamma carboxy glutamate protein 2 [BGLAP], runt-related transcription factor 2 [RUNX2], secreted phosphoprotein 1 [SPP1]) in BMSCs after METTL13 knockdown. *n* = 3. (G) Protein expression levels RUNX2 and OCN were detected by western blot. (H) Calcium nodule formation of BMSCs after METTL13 silencing detected by ARS staining. Scale bar upper: 2 μm; lower: 200 μm. *n* = 8. (I) The increased mRNA and protein expression of METTL13 after 8 days of adipogenesis induction. *n* = 3. (J) Changes of mRNA expression levels of adipogenic differentiation-related genes (ADIPOQ, CEBPα, FABP4, LPL, and PPAR-γ) after 8 days of adipogenic induction with or without METTL13 overexpression. *n* = 3. (K) Protein expression levels of PPAR-γ and FABP4. *n* = 3. (L) Lipid droplet formation level of BMSCs with or without METTL13 overexpression detected by Oil red O staining. Scale bar: 40 μm. *n* = 8. (M) Changes of mRNA expression levels of ADIPOQ, CEBPα, FABP4, LPL, and PPAR-γ after METTL13 knockdown. *n* = 3. (N) Protein expression levels of PPAR-γ and FABP4. *n* = 3. (O) Lipid droplet formation level of BMSCs with or without METTL13 knockdown detected by Oil red O staining. Scale bar: 50 μm. *n* = 8. Data are expressed as mean ± SEM. *P < 0.05; **P < 0.01; ***P < 0.001.

To confirm the role of METTL13 in BMSC differentiation, we initially determined expression during osteogenesis, detecting significant reductions in METTL13 mRNA and protein levels after 7 days of osteogenic induction (Figure 3A). Subsequently, to examine the effects of METTL13 on the osteogenic differentiation of BMSCs in vitro, we performed gain- and loss-of-function studies. After 7 days of osteogenic induction in cells overexpressing METTL13, we observed reductions in mRNA levels of the osteogenic markers ALP, BMP2, BMP4, RUNX2, and SPP1 (Figure 3B, C), along with reductions in the levels of RUNX2 and BGLAP proteins (Figure 3D), with ARS staining confirming reductions in bone mineralization (Figure 3E). Conversely, the knockdown of METTL13 enhanced the expression of these markers and contributed to an increase in matrix mineralization (Figure 3F-H). These results indicate that METTL13 negatively regulates the osteogenic differentiation of BMSCs.

Contrastingly, at both the mRNA and protein levels, we detected a significant upregulation of METTL13 after 8 days of adipogenic induction (Figure 3I). Furthermore, the experimentally induced expression of METTL13 promoted significant increases in the mRNA expression of key adipogenic marker genes, including CEBPα, FABP4, LPL, and PPAR-γ, and the expression of FABP4 and PPAR-γ proteins (Figure 3J, K). Furthermore, overexpression of METTL13 in BMSCs coincided with a significant enhancement of lipid droplet formation (Figure 3L), whereas opposite effects were observed in cells in which METTL13 had been silenced (Figure 3M-O). Collectively, these findings indicate that METTL13 drives an imbalance in BMSC differentiation, manifesting as a shift in osteogenic progression towards an adipogenic fate.

### METTL13 interacts with transcription factor Foxa1 to regulate BMSC differentiation

METTL13 is an eEF1A dimethyltransferase that enhances the production and intrinsic GTPase activity of target proteins.^20^ To establish whether the methylase activity of METTL13 is necessary for regulating the osteo-adipogenic differentiation of BMSCs, we constructed a catalytically inactive mutant, METTL13 (G58R), which was used to transfect BMSCs. Surprisingly, we found that the catalytically dead METTL13^G58R^ mutant retained its capacity to inhibit the mRNA- and protein-level expression of key osteogenesis-related genes and continued to enhance the expression of adipogenic markers in BMSCs following osteogenic or adipogenic induction (Figure S2A-D). Further verification of the expression, protein stability, and subcellular localization of the intracellular METTL13^G58R^ mutant revealed that the expression of this mutant was comparable to that of the wild-type METTL13, and that the mutant was characterized by a similar protein stability and subcellular localization (Figure S3A-C). These findings thus indicate that rather than functioning via direct enzymatic activity, METTL13 may control the fate of BMSCs through alternative molecular mechanisms.

**Figure 4.**
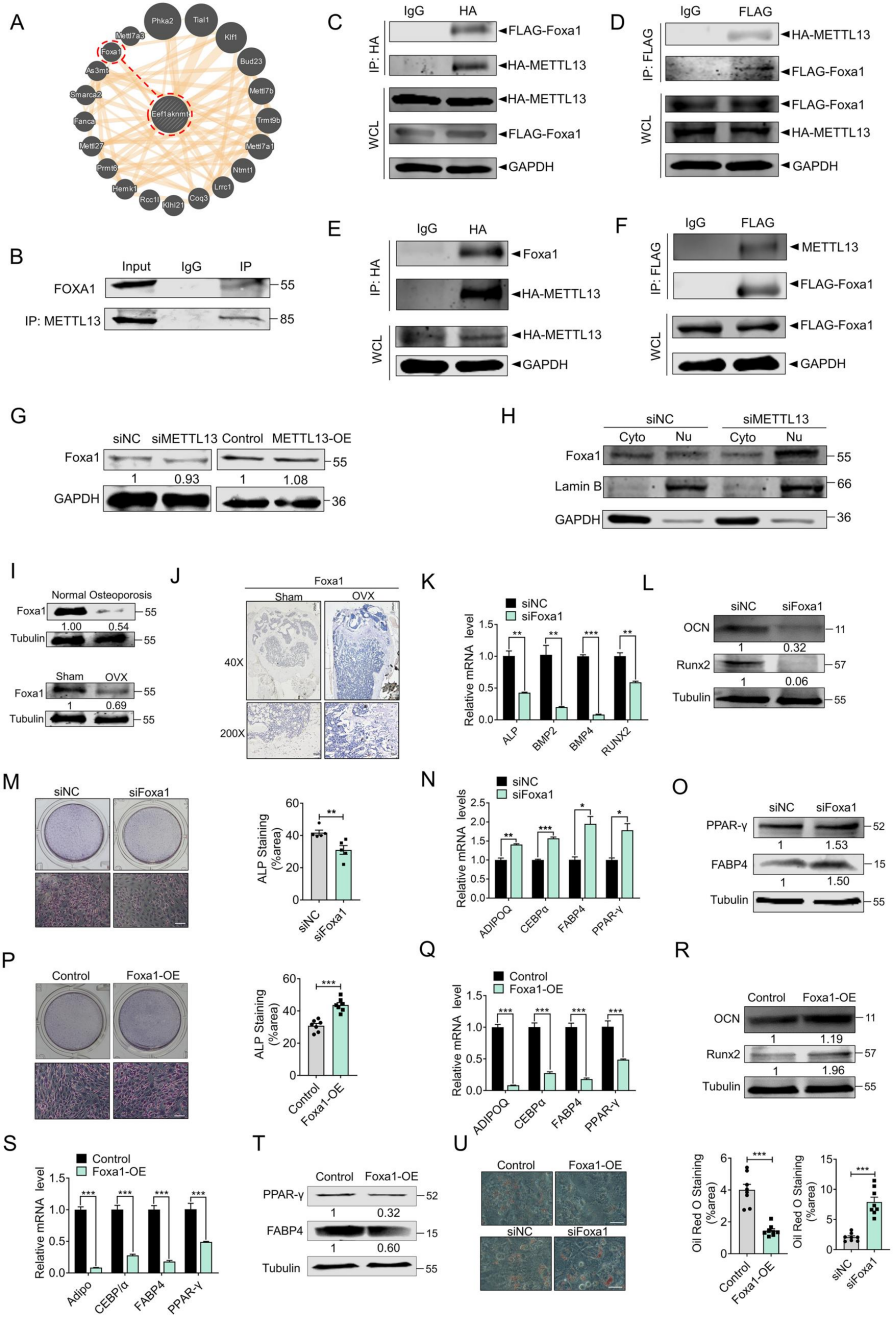
Methyltransferase-like 13 (METTL13) interacts with transcription factor forkhead box protein A1 (Foxa1) to regulate bone marrow mesenchymal stem cells (BMSCs) differentiation. (A) Prediction of METTL13 binding proteins at GeneMANIA (http://genemania). (B) Foxa1 protein expression level change in BMSCs after METTL13 silencing or METTL13 overexpression. *n* = 3. (C and D) Co-immunoprecipitation (Co-IP) of HA-METTL13 and Flag-Foxa1 in HEK-293T cells. (E) Co-IP of HA-METTTL13 and endogenous Foxa1 in BMSCs cells. (F) Co-IP of endogenous METTL13 and (Continued)Flag-Foxa1 in BMSCs cells. (G) Co-IP of endogenous METTL13 and endogenous Foxa1 in BMSCs cells. (H) Immunoblotting analysis of the distribution of Foxa1 protein in the cytoplasm and nucleus after BMSCs were transfected with METTL13 siRNAs for 24 h. (I) Protein expression of Foxa1 in the bone of normal person or osteoporosis patients and Sham or OVX mice. (J) IHC analysis of Foxa1 protein expression in the bone of Sham and OVX mice. Representative IHC images are presented. Scale bars: 200 μm (upper); 50 μm (bottom). *n* = 3. (K) Change of osteogenesis-related genes (alkaline phosphatase [ALP], bone morphogenetic protein 2 [BMP2], morphogenetic protein 4 [BMP4], runt-related transcription factor 2 [RUNX2]) mRNA expression after 7 days of osteogenic induction with or without Foxa1 knockdown. *n* = 3. (L) The protein level of OCN and RUNX2 was detected by western blot. *n* = 3. (M and N) Alkaline phosphatase levels in BMSCs cells were detected by ALP staining. Bar: 200 μm. *n* = 5. (O) Changes of mRNA expression levels of ADIPOQ, FABP4, CEBPα, and PPAR-γ after 8 days of adipogenic induction with or without Foxa1overexpression. *n* = 3. (P) Protein levels of OCN, RUNX2 with or without Foxa1 overexpression after 7 days of osteogenic induction. (Q) Changes of mRNA expression levels of ADIPOQ, FABP4, CEBPα, and PPAR-γ after 8 days of adipogenic induction with or without Foxa1 silencing. *n* = 3. (R) Protein levels of PPAR-γ and FABP4 in BMSCs after 8 days of adipogenic induction. *n* = 3. (S) Changes of mRNA expression levels of Adipo, CEBPα, FABP4, and PPAR-γ after 8 days of adipogenic induction with or without Foxa1overexpression. *n* = 3. (L) Protein levels of PPAR-γ, FABP4 in BMSCs detected by western blot. *n* = 3. (M) Lipid droplet formation level of BMSCs with Foxa1 overexpression or knockdown detected by Oil red O staining. Scale bar: 50 μm. *n* = 8. Data are expressed as mean ± SEM. *P < 0.05; **P < 0.01; ***P < 0.001.

To further assess the potential targets of METTL13, we searched the functional protein–protein interaction (PPI) network of METTL13 (Figure 4A). Notably, Foxa1, a pioneer transcription factor, was predicted to bind to METTL13. We subsequently verified the regulatory association between METTL13 and Foxa1, with Co-IP experiments confirming a strong interaction between endogenously expressed METTL13 and Foxa1 in BMSCs (Figure 4B). Furthermore, immunoprecipitation of hemagglutinin-tagged METTL13 using an anti-HA antibody was observed to pull down endogenous Foxa1 in BMSCs (Figure 4C). Conversely, in BMSCs transfected with Flag-tagged Foxa1 plasmids, we detected an interaction between exogenous Foxa1 and endogenous METTL13 (Figure 4D). Reciprocal Co-IP enabled us to further verify the interaction between ectopically expressed HA-METTL13 and Flag-Foxa1 in HEK-293T cells (Figure 4E, F). In addition, consistent with our previous findings regarding the non-enzymatic activity-dependent function of METTL13, we demonstrated that METTL13^G58R^ can bind to the Foxa1 protein (Figure S3D). However, neither the knockdown of METTL13 nor its overexpression had any significant effects on Foxa1 protein levels (Figure 4G). As a transcription factor, Foxa1 is dependent on a nuclear localization, and thus we investigated whether METTL13 regulates its nuclear translocation. METTL13 silencing increased nuclear Foxa1 levels and decreased its cytosolic expression (Figure 4H). Collectively, these results indicate that METTL13 blocks the nuclear translocation of Foxa1 via protein binding.

Given that a range of transcription factors play major regulatory roles in the differentiation of BMSCs,^25^ we hypothesized that Foxa1 regulates the BMSC differentiation. By way of verification, we examined changes in Foxa1 protein during the development of osteoporosis, which revealed that compared with normal bone, both human- and mouse-derived osteoporotic bone was characterized by significantly lower levels of Foxa1 protein (Figure 4I). Moreover, immunohistochemical staining confirmed that compared with the sham-operated mice, OVX mice had lower levels of Foxa1 protein (Figure 4J). To identify the role played by Foxa1 in the osteo-adipogenic differentiation of BMSCs, we performed gain- and loss-of-function assays followed by osteogenic or adipogenic induction in BMSCs (Figure S3A, B). Consistent with our in vitro results, Foxa1 knockdown was associated with a significantly inhibited expression of key osteogenesis-related genes (Figure 4K, L), along with a reduction in ALP activity (Figure 4M), whereas the opposite effects were observed in response to Foxa1 overexpression (Figure 4N). Further investigation of the involvement of Foxa1 in adipogenic differentiation based on qRT-PCR and western blot analyses provided confirmatory evidence that Foxa1 plays a negative regulatory role in the expression of adipogenic differentiation-related genes (Figure 4O-T). Moreover, Oil Red O staining revealed that an overexpression of Foxa1 coincided with a significant inhibition of lipid droplet formation in BMSCs, whereas Foxa1 knockdown had the opposite effects (Figure 4U). Subsequent silencing of Foxa1 was found to reverse the promotive effects of METTL13 knockdown on the expression of osteogenesis-related mRNAs and proteins, as well as the inhibitory effects of METTL13 knockdown on the expression of lipogenesis-related mRNAs and proteins (Figure S5). Consequently, silencing Foxa1 abolished the effects of knocking out METTL13. We also verified these observations in animals (Figure S6). These findings accordingly indicate that Foxa1 is required for the osteogenic differentiation of BMSCs and hampers adipogenic differentiation, which in turn retards the progression of osteoporosis.

**Figure 5.**
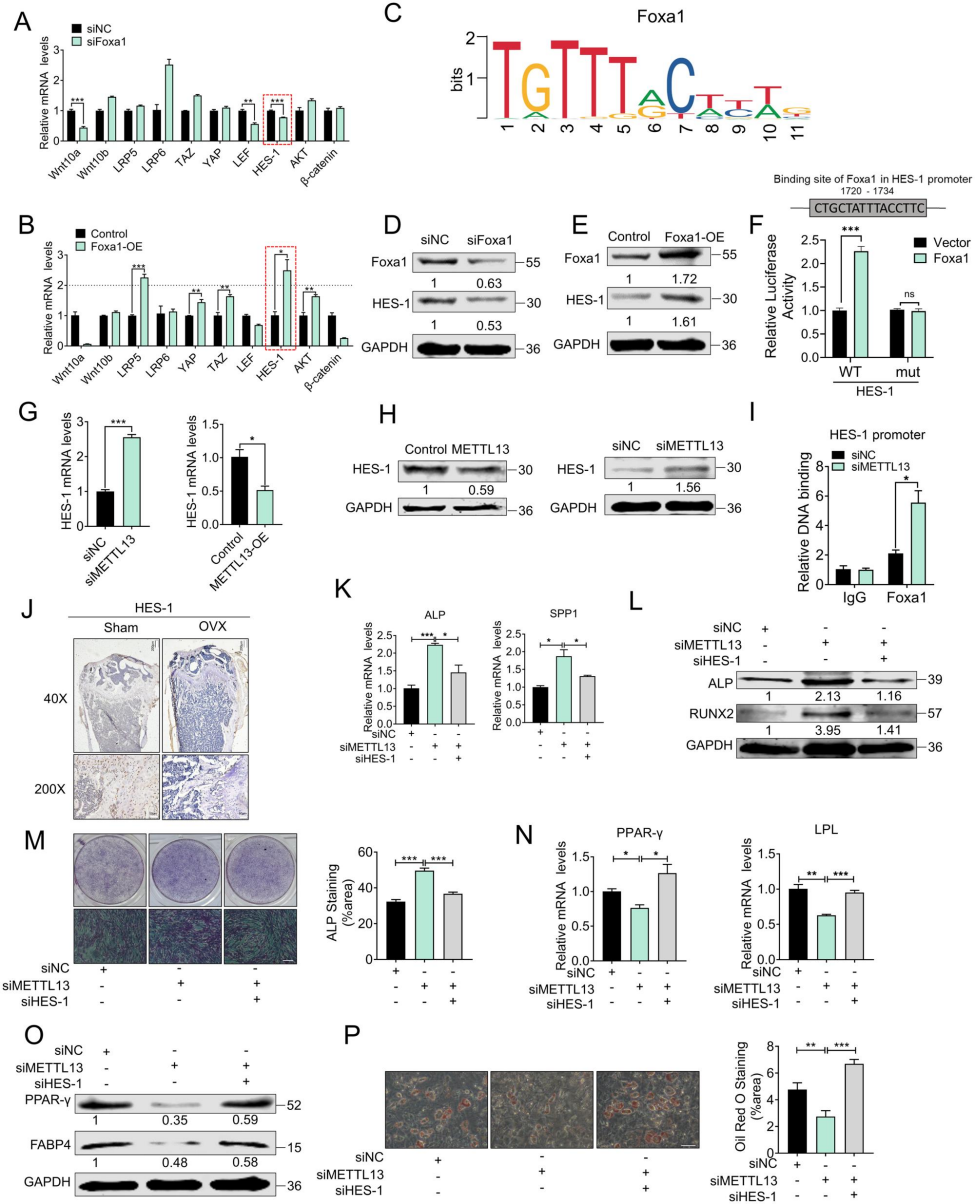
Methyltransferase-like 13 (METTL13) inhibits the transcriptional activation of forkhead box protein A1 (Foxa1) on the HES-1 promoter. (A and B) Changes of mRNA expression levels of key genes regulating osteogenic differentiation pathways (Wnt10a, Wnt10b, LRP5, LRP6, TAZ, YAP, LEF, HES-1, AKT, and β-catenin) in bone marrow mesenchymal stem cells (BMSCs) after 24 h of Foxa1 knockdown (A) or overexpression (B). *n* = 3. (C) Motifs of HES-1 enriched in Foxa1 binding sites and predicted score with JASPAR. (D and E) Protein expression of Foxa1 and HES-1 after 24 h of Foxa1 knockdown (D) or overexpression (E). *n* = 3. (F) Luciferase activity assay was performed to confirm the Foxa1 mRNA was directly bound to HES-1 promoter in HEK-293T cells. *n* = 5. (G) mRNA expression levels of HES-1 in BMSCs transfection with METTL13 siRNA or plasmids. *n* = 3. (H) Protein (Continued)expression levels of HES-1 in BMSCs transfection with METTL13 siRNA or plasmids. (I) CHIP-qPCR results show Foxa1 binding at the HES-1 promoter region with or without METTL13 silencing. *n* = 3. (J) Immunohistochemistry (IHC) analysis of Foxa1 protein expression in the bone of Sham and OVX mice. Representative IHC images of Sham and ovariectomized (OVX) are presented. Scale bars: 200 μm (upper); 50 μm (bottom). *n* = 3. (K) After 7 days of osteogenesis induction, quantitative reverse transcription-polymerase chain reaction (qRT-PCR) detected alkaline phosphatase (ALP) and secreted phosphoprotein 1 (SPP1) mRNA expression level changes after METTL13 knockdown with or without HES-1 silencing. *n* = 3. (L) Protein levels of ALP and runt-related transcription factor 2 (RUNX2) in BMSCs after 7 days osteogenesis induction. (M) ALP staining showing the ALP level of BMSCs cells after 7 days of osteogenesis induction. Bar: 200 μm. *n* = 5. (N) mRNA expression levels of PPAR-γ and LPL after 8 days of adipogenesis induction in BMSCs transfected with METTL13 siRNA or co-transfected with HES-1 siRNA. *n* = 3. (O) Protein expression levels of PPAR-γ and FABP4 after 8 days of adipogenesis induction. (P) Lipid droplet formation level of BMSCs with METTL13 knockdown with or without HES-1 silencing detected by Oil red O staining. Scale bar: 50 μm. *n* = 6. Data are expressed as mean ± SEM. *P < 0.05; **P < 0.01; ***P < 0.001.

**Figure 6.**
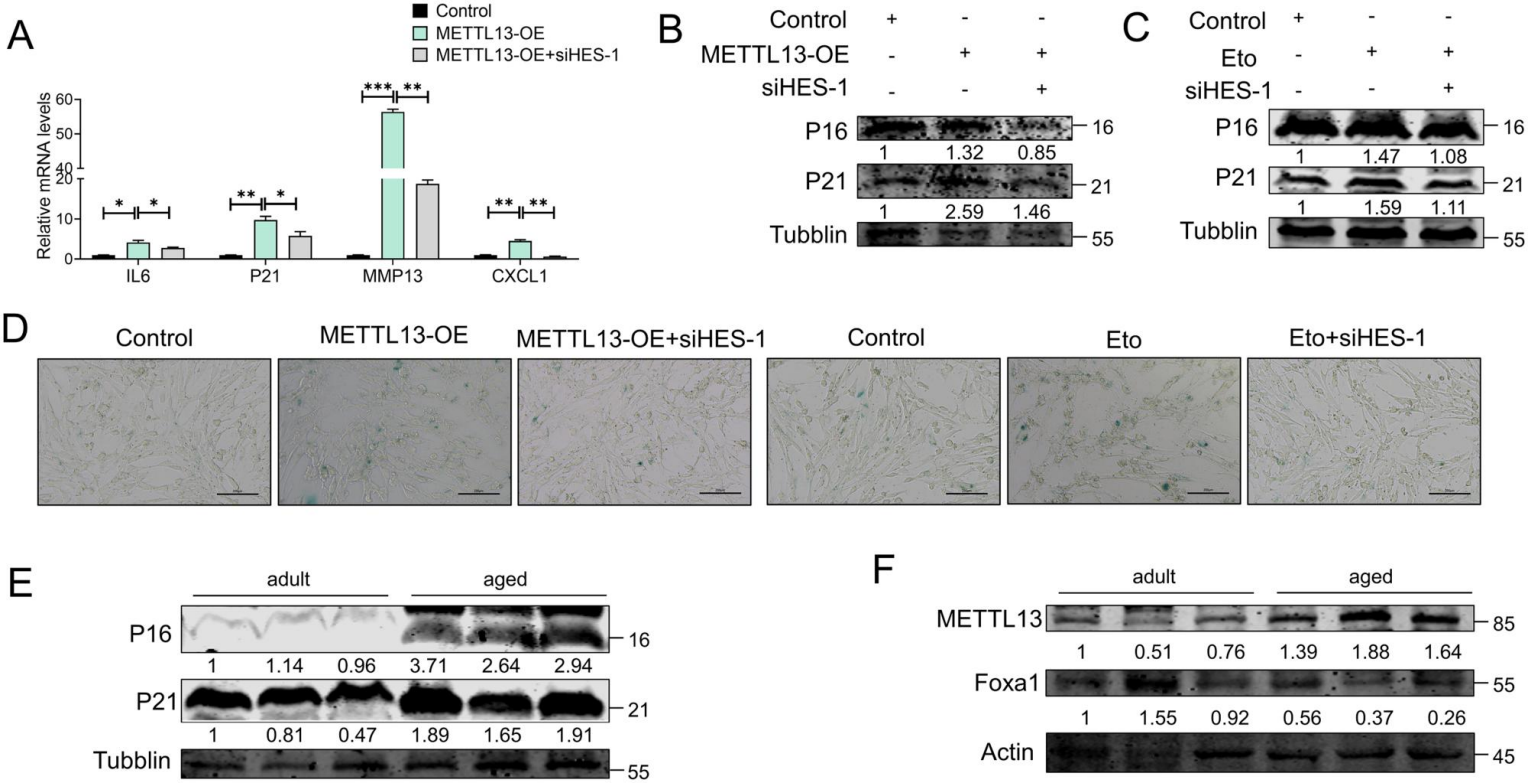
Methyltransferase-like 13 (METTL13) regulate bone marrow mesenchymal stem cells (BMSCs) senescence via forkhead box protein A1 (Foxa1)/HES-1 axis. (A) mRNA expression levels of IL-6, P21, MMP13, and CXCT1 in BMSCs transfected with METTL13 plasmid or co-transfected with HES-1 siRNA. *n* = 3. (B) Protein expression levels of P16 and P21 in BMSCs transfected with METTL13 plasmid or co-transfected with HES-1 siRNA. (C) Protein expression levels of P16 and P21 in BMSCs treated by Eto or transfected with HES-1 siRNA. (D) Beta-galactosidase staining in BMSCs with or without 5 μM etoposide treatment for 48 h. SA-β-gal-positive BMSCs are blue. Scale bar: 200 μm. (E) Protein expression levels of P16, P21, METTL13, and Foxa1 in 1.5- and 16-month-old mice respectively. Data are expressed as mean ± SEM. *P < 0.05; **P < 0.01; ***P < 0.001.

### METTL13 inhibits the transcriptional activation of Foxa1 on the HES-1 promoter

To identify putative Foxa1 transcriptional targets in BMSCs, we focused on key genes in major osteo-adipogenic regulatory pathways, including WNT, NOTCH, Hippo, and AKT signaling pathways.^3^^,^^26^ Thereafter, we performed qRT-PCR to confirm changes in the key regulatory genes in these pathways, including Wnt10a, Wnt10b, LRP5, LRP6, and LEF (WNT pathway); YAP, TAZ, β-catenin, and HES-1 (NOTCH pathway); and AKT (AKT pathway). Knockdown of Foxa1 resulted in a significant inhibition of the mRNA expression of Wnt10a, LEF, and HES-1 (Figure 5A), whereas overexpression of Foxa1 coincided with an upregulation of LRP5, YAP, TAZ, HES-1, and AKT mRNAs (Figure 5B). Among these, we found that the transcription of HES-1, an important NOTCH pathway target gene, was significantly positively regulated by Foxa1. Furthermore, by searching the JASPAR database (https://jaspar.genereg.net/analysis), we identified Foxa1 transcription factor binding sites in the DNA sequence of this protein (Figure 5C). Consistently, we demonstrated that silencing of Foxa1 contributed to reductions in HES-1 protein levels, whereas the experimentally induced expression of Foxa1 had the opposite effects (Figure 5D, E). We subsequently constructed a luciferase reporter plasmid containing the wild-type HES-1 promoter region encompassing the predicted Foxa1 binding site, along with a mutant version in which this binding site had been subjected to site-directed mutagenesis. Co-transfection of cells with Foxa1 significantly enhanced luciferase activity driven by the wild-type HES-1 promoter compared with that in the empty vector control (Figure 5F). Importantly, this activation was completely abolished when the predicted Foxa1 binding site was mutated. Accordingly, these results provide direct evidence indicating that Foxa1 transcriptionally activates HES-1 via the identified binding site. Focusing on the relationship between Foxa1 and METTL13, we further examined whether METTL13 influences the transcriptional activity of Foxa1 and found that knockdown of METTL13 promoted significant increases in the mRNA and protein levels of HES-1, whereas METTL13 overexpression produced the opposite effects (Figure 5G, H). Notably, ChIP-qPCR results revealed that the binding between Foxa1 and the HES-1 promoter was enhanced in response to the silencing of METTL13 (Figure 5I). Collectively, these findings revealed that binding of METTL13 blocks the transcriptional activation of Foxa1 at the HES-1 promoter. Thus, given our assumption that METTL13 suppresses HES-1 expression by binding to Foxa1, we further examined the role of HES-1 in METTL13-mediated BMSC differentiation. In line with recent findings indicating that HES-1 inhibits adipogenic differentiation and is expressed during osteogenic differentiation,^27^^,^^28^ immunohistochemical staining in this study revealed that compared with that in sham-operated mice, HES-1 expression was markedly reduced in the bone tissues of OVX mice (Figure 5J). Furthermore, qRT-PCR analysis revealed that knockdown of HES-1 suppressed the expression of osteogenesis-related genes (ALP and SPP1) induced by the silencing of METTL13, as well as the protein levels of ALP and RUNX2 (Figure 5K, L). In addition, ALP activity promoted by METTL13 silencing was eliminated by HES-1 knockdown (Figure 5M), indicating that HES-1 is required for the osteogenic differentiation of BMSCs mediated by METTL13 knockdown. However, we also found that HES-1 knockdown reversed the inhibition of adipogenic differentiation regulated by the silencing of METTL13 (Figure 5N-P). Finally, we extracted bone tissue proteins from OVX mice that had been injected with the AAV9-control and AAV9-shMETTL13 constructs. Consistent with our previous observations, we found that METTL13 promotes the nuclear translocation of Foxa1. Accordingly, although METTL13 knockdown in mice had no substantial effects on Foxa1 protein levels, it did induce the expression of HES-1 protein (Figure S7). To further establish involvement of the METTL13–Foxa1–HES-1 axis, we examined the effects of knocking down HES-1 on the osteogenic effects associated with Foxa1 overexpression, the findings of which revealed that HES-1 knockdown reversed the promotive effects of elevated levels of Foxa1 on the expression of osteogenic-related RNAs and proteins (Figure S8). These findings thus provided evidence that the role of METTL13 in controlling BMSC differentiation may be mediated, at least partially, by binding to Foxa1, which leads to an impairment in the transcriptional activation of HES-1.

### METTL13 regulates BMSC senescence via the Foxa1/HES-1 axis

Our examination of aging-related genes in mesenchymal stem cells revealed that knocking down HES-1 reversed the aging-related effect promoted METTL13 overexpression (Figure 6A), with consistent results being obtained at the protein level (Figure 6B). Moreover, we established that knockdown of HES-1 also reversed the increase in aging-related proteins promoted by etoposide (Figure 6C), with the results of β-galactosidase staining further confirming our hypothesis (Figure 6D). Subsequent analysis of proteins in bone tissues extracted from 1.5- and 16-month-old mice confirmed that the aging model had been successfully established (Figure 6E). In these aging mice, we detected significantly elevated levels of METTL13 protein, whereas the expression of Foxa1 was notably reduced (Figure 6F). Given these findings, we thus conclude that METTL13 mediates mesenchymal stem cell aging via Foxa1 and HES-1, ultimately regulating the progression of osteoporosis.

## Discussion

Estrogen deficiency associated with aging is among the primary factors contributing to the development of post-menopausal osteoporosis.^29^ Although the combined effects of aging-induced cell senescence and BMSC differentiation in the development of osteoporosis have generated considerable interest, the underlying molecular mechanisms have yet to be sufficiently elucidated. In this study, we identified METTL13, an eEF1A methyltransferase, as a regulator of bone mass that functions by promoting cell senescence and controlling the lineage commitment of BMSCs. The expression of METTL13 was established to be positively correlated with the aging process of BMSCs and found to exacerbate osteoporosis in OVX mice. Furthermore, we demonstrated that by binding to the transcription factor Foxa1, METTL13 regulates the osteo-adipogenic balance, thereby negatively regulating the expression of HES-1.

We have previously discovered that by targeting BMSC differentiation, m6A methyltransferases play vital roles in the development of osteoporosis.^8^^,^^18^ METTL13 was originally identified as a candidate eEF1A lysine 55 dimethyltransferase that triggers eEF1A GTPase activity and cellular mRNA translation,^19^^,^^30^ and emerging evidence indicates that METTL13 plays essential roles in tumorigenesis and cancer progression.^20^^,^^23^^,^^31^ Notably, variants of METTL13 have also been identified in cases of human GAB1-associated profound deafness, further highlighting the importance of METTL13 in the context of human diseases.^32^ However, as to whether METTL13 is involved in the fate of stem cells has hitherto remained undetermined. In this study, we showed that in both humans and mice, the expression of METTL13 is upregulated during osteoporosis, whereas downregulation of METTL13 expression prevents the progression of this bone loss. Importantly, we established that METTL13 induces cell senescence, which further promotes the adipogenic differentiation of BMSCs and suppresses osteogenic differentiation.

Previous studies have identified strong associations between lysine methyltransferases and the differentiation of BMSCs. For example, it has been established that enhancer of Zeste homolog 2, which methylates histone H3 at lysine 27, shifts mesenchymal stem cell lineage commitment towards adipocytes rather than osteoblasts, thereby promoting the progression of osteoporosis.^30^^,^^33^ Surprisingly, we found that supplementation with the catalytically inactivated METTL13^G58R^ similarly reduced osteogenic differentiation, whilst promoting adipogenic differentiation. This accordingly provides evidence that the mechanisms whereby METTL13 determines the adipogenic fate of BMSCs probably occur independently of the conventional type of methylation, potentially via a novel mechanism of action. PPI networks are among the most important cellular biochemical networks, the dysregulation of which is commonly cited as a cause of disorders associated with stem cell differentiation.^34^ Thus, with a view towards identify the targets of METTL13, we searched a functional PPI network of METTL13, based on which, the transcription factor Foxa1 was predicted to bind to the METTL13 protein.^35–37^

It is well established that osteoblast differentiation involves a range of transcription factors, which are accordingly considered excellent candidate genes for controlling the differentiation of BMSCs. Among these factors, Foxa1, an important member of the forkhead/winged-helix transcription factor family, has been reported to play roles in the differentiation of multiple stem cell types, including pluripotent, adipose-derived, and embryonic stem cells.^38–40^ Foxa1 functions as a pioneer transcription factor that regulates the binding of other transcription factors to target genes, thereby influencing the expression of downstream targets.^41^ Evidence indicates that the microRNA miR-4721 directly targets Foxa1 and activates Nanog and downstream regulators of stem cell signaling, thereby promoting neural progenitor cell enrichment and metastasis.^42^ However, the association between Foxa1 and BMSC differentiation has yet to be determined. In this study, we have demonstrated that when experimentally induced, Foxa1 can promote the osteogenic differentiation of BMSCs, whereas a depletion of Foxa1 coincides with a suppression of this differentiation, with the converse effects being observed with respect to adipogenic differentiation. Moreover, we reveal that METTL13 mediates BMSC differentiation by promoting the nuclear translocation of Foxa1. Consequently, these findings indicate that the combined activities of METTL13 and Foxa1 contribute to regulating the balance of osteo-adipogenic differentiation in BMSCs.

Nevertheless, although our findings provide strong evidence for the involvement of METTL13 in regulating adipogenic and osteogenic differentiation in BMSCs, we also acknowledge that this study has certain limitations. Firstly, evidence regarding the non-enzymatic function of METTL13 remains limited. Although we generated an enzyme activity-deficient METTL13 mutant via a point mutation and evaluated its effects on osteo-adipogenic differentiation in BMSCs in vitro and in cell experiments, both of which indicated the occurrence of non-enzymatic mechanisms, the current evidence is mainly based on correlation and loss-of-function phenotypes. Thus, we cannot completely rule out the influence of residual enzymatic activity or complex in vivo feedback regulation. Future studies should therefore adopt more refined approaches, such as developing next-generation inhibitors that can specifically block the enzymatic activity of METTL13, or using knock-in mouse models to introduce mutations that completely abolish activity, which could clearly contribute to distinguishing the enzymatic and non-enzymatic functions of METTL13 under more relevant physiological conditions. Secondly, although the mouse OVX model used in this study effectively addresses our preliminary hypothesis regarding the regulation of osteoporosis by METTL13, it may not fully recapitulate the complexity of human diseases. Consequently, we intend to examine the function of METTL13 in osteoporotic mice using aging models. However, these limitations do not undermine our main conclusions, but instead provide a clear roadmap for future research directions, which will contribute to a more comprehensive and rigorous clarification of the biological function of METTL13.

Accumulating evidence has revealed that certain essential signaling pathways, including the Hippo, WNT, NOTCH, and AKT pathways, are involved in governing the lineage commitment of BMSCs and may shift the balance from osteogenesis towards adipogenesis.^43–46^ Among the key regulators of these classical pathways, we found that the expression HES-1 was characterized by the most pronounced changes in response to either the overexpression or silencing of METTL13. As a key downstream target of the NOTCH signaling pathway, HES-1 may regulate the differentiation and function of osteoblasts and osteoclasts involved in bone remodelling.^47^ Suitably manipulating modulators of the NOTCH pathway to mediate osteogenesis could thus represent a viable strategy for treating bone diseases such as osteoporosis.^48^ Indeed, it has been reported that a disruption of NOTCH signaling in mice leads to osteopenia, long bone shortening, and bone loss, whereas an overexpression of HES-1 in mesenchymal stem cells has been demonstrated to block the expression of PPAR-γ and CEBPα, inhibit adipogenic differentiation, and promote osteogenic differentiation.^49^ Consistently, we show that HES-1 expression is negatively correlated with the development of osteoporosis, and that knockdown of METTL13 enhances the binding of Foxa1 to the HES-1 promoter. Furthermore, the silencing of HES-1 was found to substantially reverse the inhibitory or promotive effects of METTL13 knockdown on osteogenic and adipogenic differentiation, thereby providing evidence that METTL13-mediated BMSC differentiation is attributable, in part at least, to an inhibition of Foxa1 transcriptional activity, which contributes to a further reduction in HES-1 transcription.

In this study, we thus established the pivotal involvement of the METTL13–Foxa1–HES-1 axis in regulating osteoporosis. Although based largely on murine models, our findings provide sufficient evidence to indicate that focusing on this regulatory system represents an important direction for the future development of clinical treatments for osteoporosis.

## Conclusions

We provide compelling in vitro and in vivo evidence to indicate that METTL13 plays a central role in determining the progression of osteoporosis through its influence on BMSC aging and in mediating the osteo-adipogenic balance of BMSCs via the Foxa1/HES-1 axis. Moreover, our findings will provide a basis for identifying novel biomarkers of osteoporosis and developing therapeutic approaches targeting METTL13.

## Supplementary Material

szag049_Supplementary_Data

## Data Availability

Data supporting the findings of this study are available from the corresponding author upon request.
