# Supplementary material for: Lysine methyltransferase methyltransferase-like 13 regulates bone marrow mesenchymal stem cells osteo-adipogenic differentiation and senescence in osteoporosis via the Foxa1/HES-1 axis
Source: Stem Cells Transl Med. 2026 Jul 28;15(8):szag049. doi: 10.1093/stcltm/szag049 (PMC13415456; doi:10.1093/stcltm/szag049)
Supplement: szag049_Supplementary_Data [file szag049_supplementary_data.docx]

**Supplementary Information**

**Lysine methyltransferase METTL13 regulates BMSCs osteo-adipogenic differentiation and senescence in osteoporosis via the Foxa1/HES-1 axis**Ying Liu^1*^, Ao Wang^1*^, Ziwen Liu^2*^, Zhengwei Qin ^3,4^, Lei Yin ^2,3^, Xinyue Wang^1^, Mingyu He^1^, Tao Li^1^, Yanquan Wang^1,5^, Longhao Chen^1^, Zhuo Liu^1^, Jiayi Liu^3,4^, Binsha Gu^3,4^, Shichao Yang^3,4^, Hui Liang^1^, Yang Li^6,7^, Xiaofei Zheng^8^, Shuixing Zhang^9^, Baofeng Yang^1#^, Ye Yuan^3#^, Lei Yang^5,10,11#^

^1^Department of Pharmacology (State Key Laboratory of Frigid Zone Cardiovascular Diseases (SKLFZCD), State-Province Key Laboratories of Biomedicine Pharmaceutics of China, Key Laboratory of Cardiovascular Research, Ministry of Education), College of Pharmacy, Harbin Medical University, Harbin, China.

^2^Department of Orthopedic Surgery, The Second Affiliated Hospital of Harbin Medical University, Harbin, Heilongjiang, China.

^3^Department of Pharmacy at the Second Affiliated Hospital, State Key Laboratory of Frigid Zone Cardiovascular Diseases (SKLFZCD), Harbin Medical University, Harbin, China.

^4^Department of Clinical Pharmacology, College of Pharmacy, Harbin Medical University, Harbin, China.

^5^Department of Orthopedics at the First Affiliated Hospital, State Key Laboratory of Frigid Zone Cardiovascular Diseases (SKLFZCD), Harbin Medical University, Harbin, China.

^6^Center for Endemic Disease Control, Chinese Center for Disease Control and Prevention, Harbin Medical University, Harbin, 150081, China.

^7^NHC Key Laboratory of Etiology and Epidemiology (Harbin Medical University), Joint Key Laboratory of Endemic Diseases（Harbin Medical University；Guizhou Medical University；Xi'an Jiaotong University), Heilongjiang Provincial Key Laboratory of Trace Elements and Human Health; Key Laboratory of Etiology and Epidemiology, Education Bureau of Heilongjiang Province, Harbin Medical University, Harbin, 150081, China.

^8^Department of Sports Medicine, The First Affiliated Hospital, Guangdong Provincial Key Laboratory of Speed Capability, The Guangzhou Key Laboratory of Precision Orthopedics and Regenerative Medicine, School of Medicine, Jinan University.

^9^Department of Radiology, The First Affiliated Hospital of Jinan University, Guangzhou, Guangdong, China.

^10^Key Laboratory of Hepatosplenic Surgery of Ministry of Education, The First Affiliated Hospital of Harbin Medical University, Harbin, China.

^11^NHC Key Laboratory of Cell Transplantation, The First Affiliated Hospital of Harbin Medical University, Harbin, China.

*Equal contributors to this work

^#^Corresponding Author

**Correspondence to:**

Prof. Lei Yang, Department of Orthopedics at the First Affiliated Hospital, State Key Laboratory of Frigid Zone Cardiovascular Diseases (SKLFZCD), Harbin Medical University, Harbin, China; [yangray83@vip.qq.com](mailto:yangray83@vip.qq.com);

Dr. Ye Yuan, Department of Pharmacy at the Second Affiliated Hospital, State Key Laboratory of Frigid Zone Cardiovascular Diseases (SKLFZCD), Harbin Medical University, Harbin, China; [yuanye_hmu@126.com](mailto:yuanye_hmu@126.com);

Prof. Baofeng Yang, Department of Pharmacology, State Key Laboratory of Frigid Zone Cardiovascular Diseases (SKLFZCD), Harbin Medical University, Harbin, China; [yangbf@ems.hrbmu.edu.cn](mailto:yangbf@ems.hrbmu.edu.cn) .


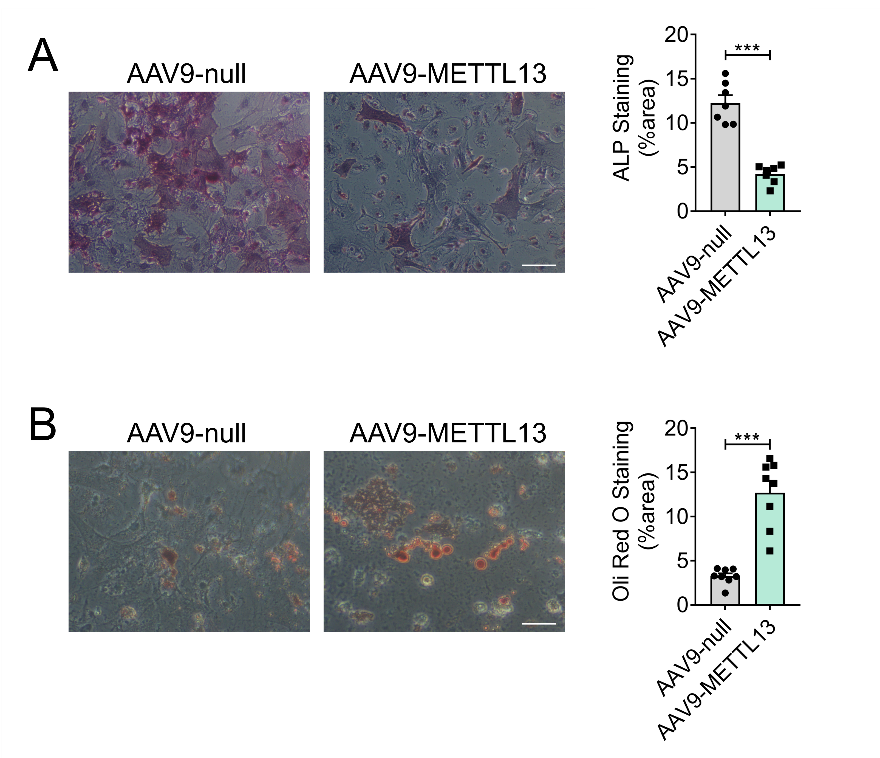
**Figure S1**

**Figure S1.** **Primary BMSCs from METTL13 overexpressed mice had decreased osteogenic differentiation ability and increased lipid differentiation ability.** (A) ALP level in primary BMSCs detected by ALP staining. Bar: 100 μm. n=7. (B) Oil red O staining showed the lipid droplet formation level of primary BMSCs. Bar: 200 μm. n=8. Data are expressed as mean ± SEM. ***P < 0.001.


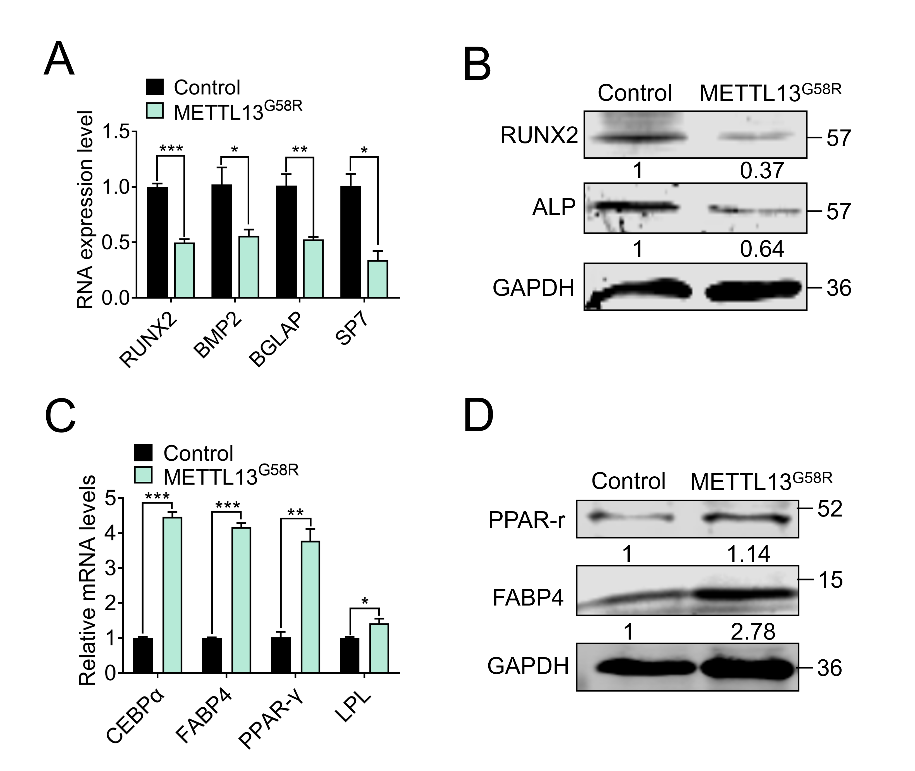
**Figure S2**

**Figure S2. Mutation of METTL13 (METTL13^G58R^) inhibits osteogenic differentiation and promotes adipogenic differentiation of BMSCs.** (A) mRNA expression levels of RUNX2, BMP2, BGLAP and SP7 after 7 days osteogenesis induction in BMSCs transfected with METTL13^G58R^ plasmid. n=3. (B) Protein expression level of RUNX2 and ALP after 7 days osteogenesis induction in BMSCs. (C) mRNA expression levels of CEBP/α，FABP4, PPAR-γ and LPL after 8 days adipogenesis induction in BMSCs transfected with METTL13^G58R^ plasmid. n=3. (D) Protein expression levels of PPAR-γand FABP4 in BMSCs. Data are expressed as mean ± SEM. *P < 0.05; **P < 0.01; ***P < 0.001.

**Figure S3**


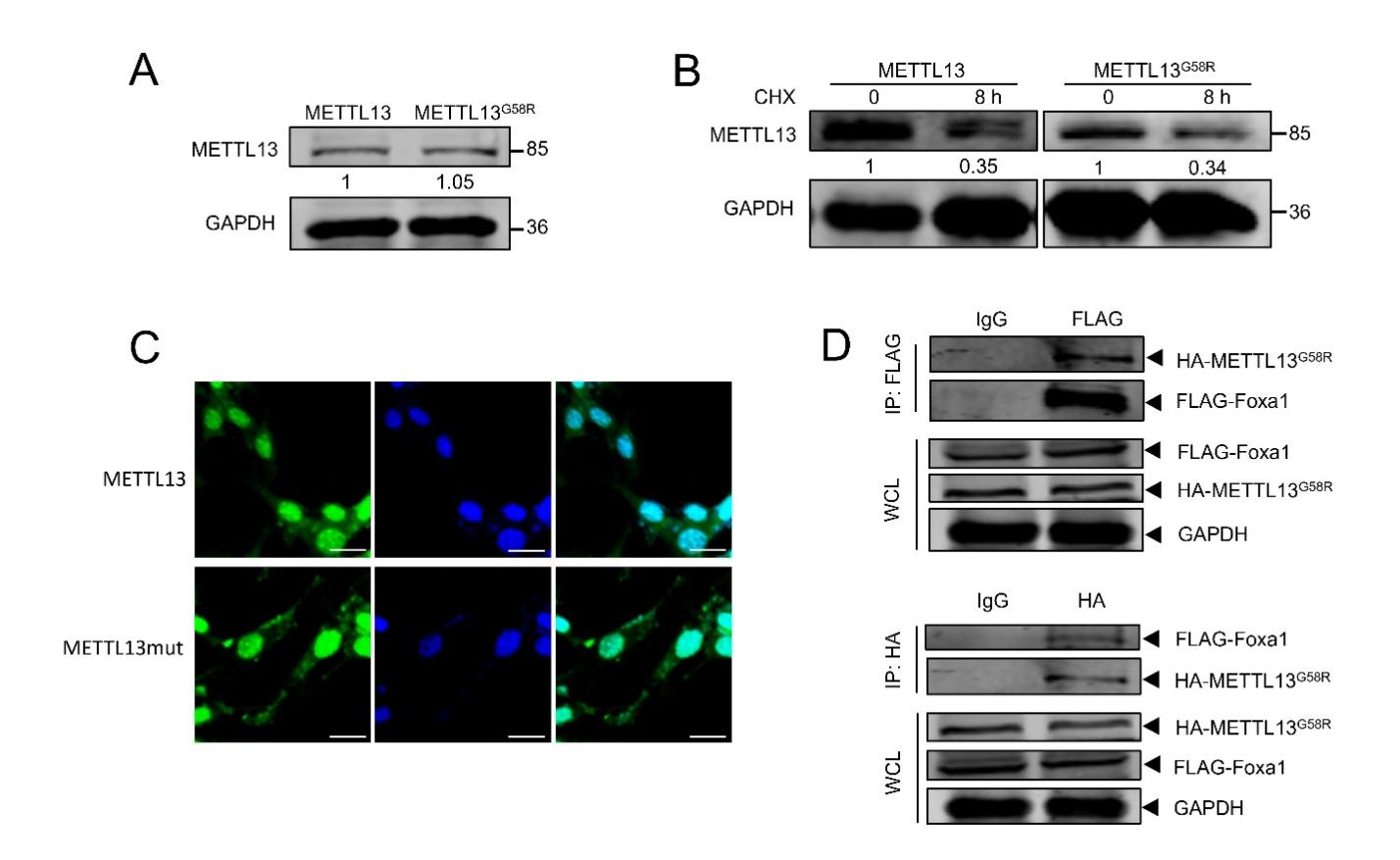
**Figure S3. The basic characterization of the METTL13^G58R^ is consistent with that of the wild-type METTL13.** (A) Protein expression level of METTL13 after transfecting with equal amounts of the METTL13^G58R^ plasmid and the wild-type METTL13 plasmid in BMSCs. (B) After transfecting equal amounts of METTL13^G58R^ and wild-type METTL13 plasmids into BMSC, CHX (100 ug/ml) was added and the METTL13 protein levels were measured 0 hours and 8 hours later. (C) The immunofluorescence experiment revealed the intracellular localization of METTL13 and METTL13^G58R^. Bar: 50 μm. n=5. (D) The Co-IP experiment was used to detect the binding of the METTL13^G58R^ protein to the Foxa1 protein. Data are expressed as mean ± SEM.


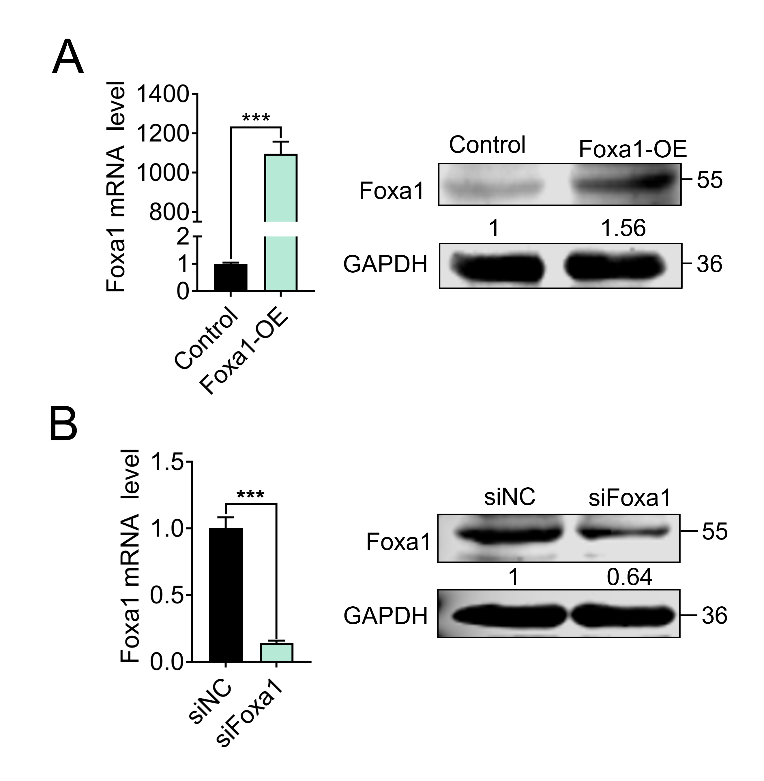
**Figure S4**

**Figure S4. Transfection efficiency of Foxa1 overexpression or knockdown.** (A) Foxa1 overexpression (A) or knockdown (B) efficiency confirmed by qRT-PCR and western blot. Data are expressed as mean ± SEM. ***P < 0.001.

**Figure S5**

**
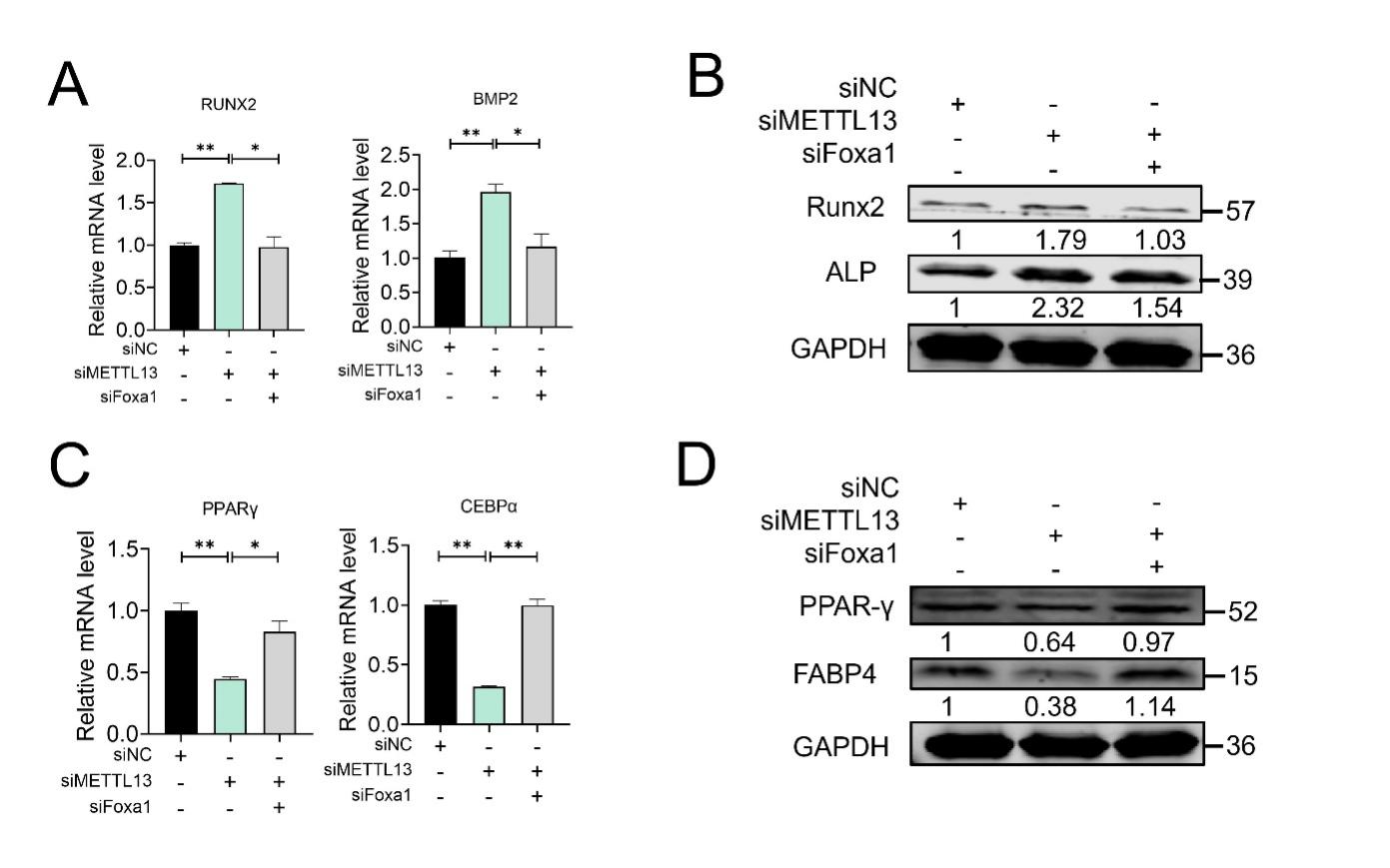
**

**Figure S5. Knocking down of Foxa1 reversed the effect of knocking down of METTL13 on inhibiting osteogenic differentiation while promoting adipogenic differentiation of BMSCs.** (A) mRNA expression levels of RUNX2 and BMP2 after 7 days osteogenesis induction in BMSCs transfected with siMETTL13 and siFoxa1. n=3. (B) Protein expression level of RUNX2 and ALP after 7 days osteogenesis induction in BMSCs. (C) mRNA expression levels of PPAR-γ and CEBP/α after 8 days adipogenesis induction in BMSCs transfected with siMETTL13 and siFoxa1. n=3. (D) Protein expression levels of PPAR-γ and FABP4 in BMSCs. Data are expressed as mean ± SEM. *P < 0.05; **P < 0.01.

**Figure S6**


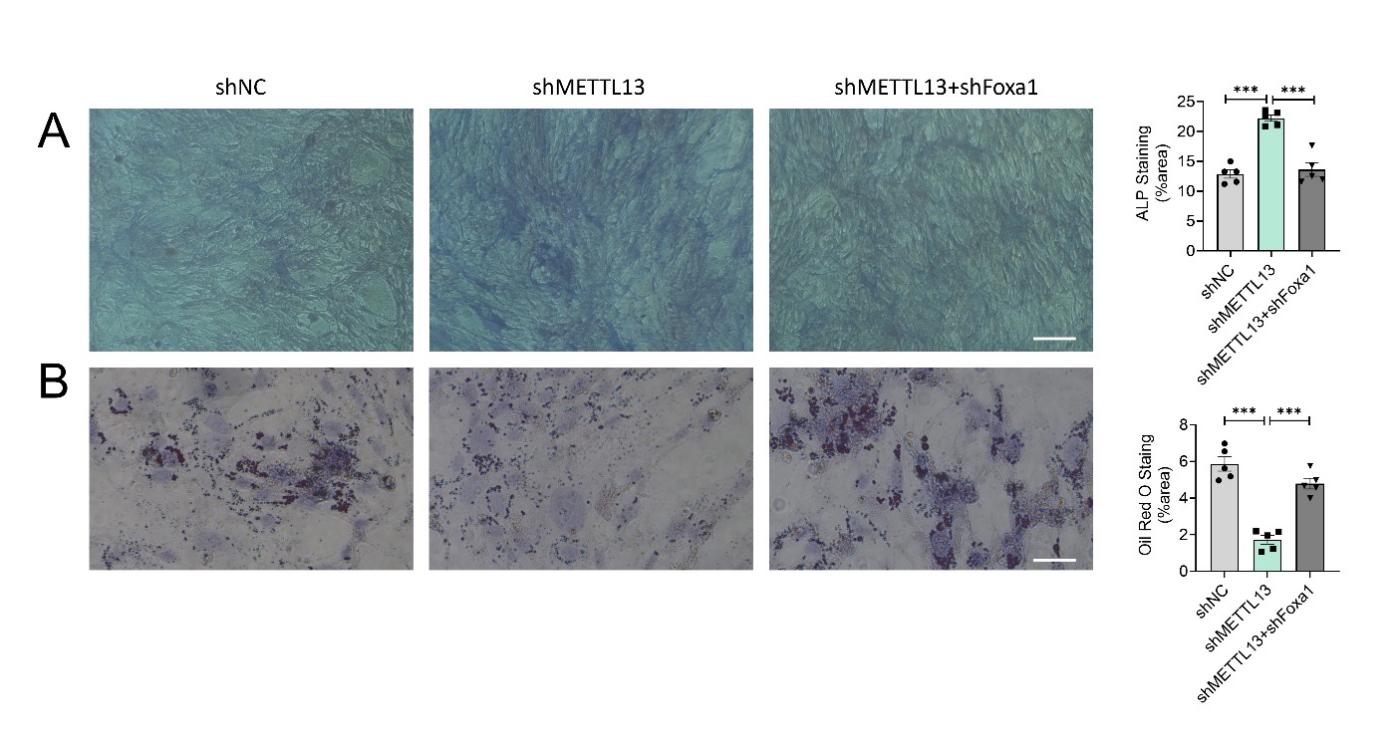


**Figure S6.** **Primary BMSCs from Knocking down Foxa1 mice reversed the ability of METTL13 knockdown to promote osteogenic differentiation and increased lipid differentiation ability.** (A) ALP level in primary BMSCs detected by ALP staining. Bar: 100 μm. n=7. (B) Oil red O staining showed the lipid droplet formation level of primary BMSCs. Bar: 200 μm. n=8. Data are expressed as mean ± SEM. ***P < 0.001.

**Figure S7**

**
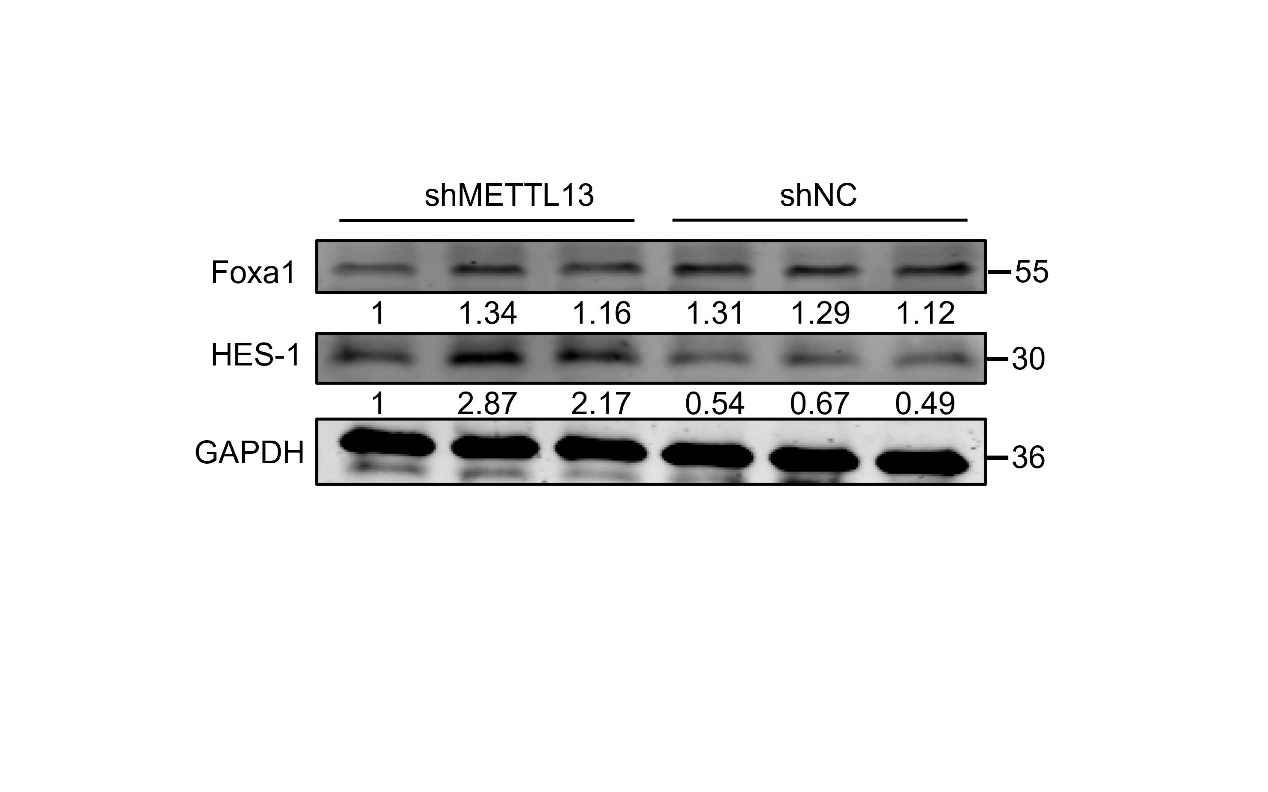
 Figure S7.** **Knocking out METTL13 in mice does not affect the protein level of Foxa1 in bone tissue, but it increases the protein level of HES-1.** Protein expression level of Foxa1 and HES-1 from bone tissue in knocking out METTL13 mice .

**Figure S8**


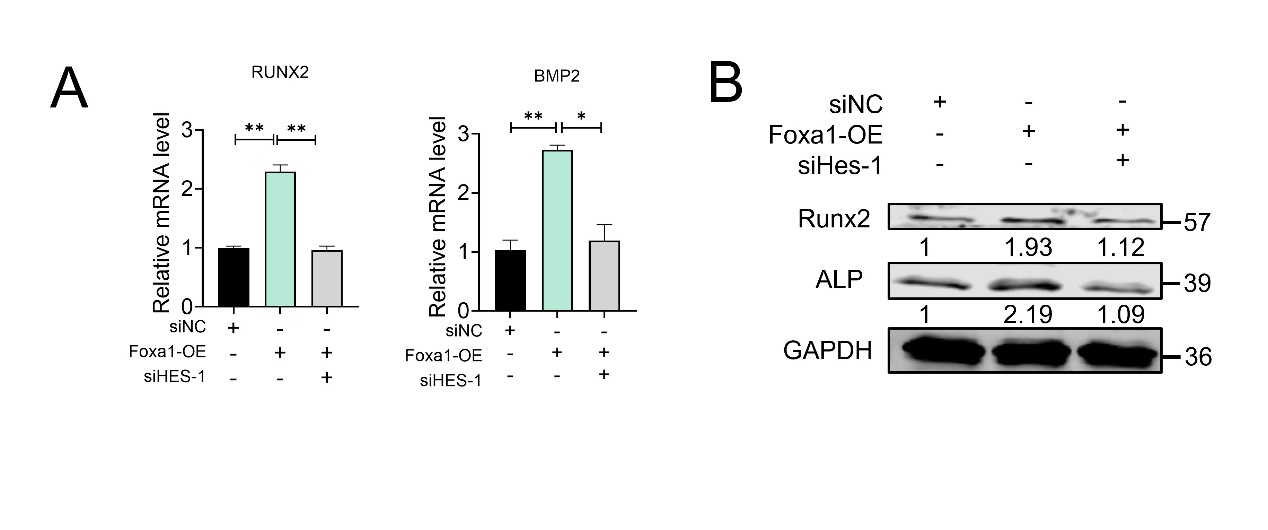
 **Figure S8. Knocking down of HES-1 reversed the effect of overexpression of Foxa1 on promoting osteogenic differentiation of BMSCs.** (A) mRNA expression levels of RUNX2 and BMP2 after 7 days osteogenesis induction in BMSCs transfected with Foxa1-OE and siHES-1. n=3. (B) Protein expression level of Runx2 and ALP after 7 days osteogenesis induction in BMSCs Data are expressed as mean ± SEM. *P < 0.05; **P < 0.01.

**Table S1: the primer pairs used in our qRT-PCR analysis.**

| **Primer** | **Forward primer sequence (5`-3`)** | **Reverse primer sequence (5`-3`)** |
| --- | --- | --- |
| m-ALP | GCCTGGATCTCATCAGTATTTGG | GTTCAGTGCGGTTCCAGACAT |
| m-AKT | CCTGAAGCTGGAGAACCTCA | TTCATAGTGGCACCGTCCTT |
| m- ADIPQ | CCTCTTAATCCTGCCCAGTC | TCCTGTCTCACCCTTAGGAC |
| m-BGLAP | CTCTGTCTCTCTGACCTCACAG | CAGGTCCTAAATAGTGATACC |
| m-BMP2 | CCTTGCTGACCACCTGAACT | AACATGGAGATTGCGCTGA |
| m-BMP4 | TCGTTACCTCAAGGGAGTGG | ATGCTTGGGACTACGTTTGG |
| m-CEBPα | CTCGCTCCTTTTCCTACCG | AGGAAGCAGGAATCCTCC |
| m-FABP4 | AAATCACCGCAGACGACA | CACATTCCACCACCAGCT |
| m-Foxa1 | ACATTCAAGCGCAGCTACCC | TGCTGGTTCTGGCGGTAATAG |
| m-HES-1 | TGCCCGGAAGGATTGACC | TGCCAGGATGTTTTATTGC |
| m-LRP5 | ACGTCCCGTAAGGTTCTCTTC | GCCAGTAAATGTCGGAGTCTAC |
| m-LRP6 | CTGCGGTGGACTTTGTGTTT | ATCGGCCCAGTAAAGCTTCC |
| m-LEF1 | CCAACTTTCCGGAGGAGGC | GTAGGAGGGTCCCTTGTTGTAC |
| m-LPL | TCTCCTGATGACGCTGATTTTG | TCTCTTGGCTCTGACCTTGTTG |
| m-METTL13 | CCTGCAGGTGCCCTTCC | CCGTTTCTTCTGGGCTCTGT |
| m- PPAR-γ | TGGGTGAAACTCTGGGAGATTC | AGAGGTCCACAGAGCTGATTCC |
| m-RUNX2 | ACTTCCTGTGCTCCGTGCTG | TCGTTGAACCTGGCTACTTGG |
| m-SPP1 | ACACTTTCACTCCAATCGTCC | TGCCCTTTCCGTTGTTGTCC |
| m-SP7 | ATGGCGTCCTCTCTGCTTG | TGAAAGGTCAGCGTATGGCTT |
| m-TAZ | GCAGACATCTGCTTCACCAA | TGAAGTCCATCCCTTTCTGG |
| m-Wnt10a | CAGATCGCCATCCATGAGTG | ACCGCAAGCCTTCAGTTTACC |
| m-Wnt10b | GCGGGTCTCCTGTTCTTGG | CCGGGAAGTTTAAGGCCCAG |
| m-YAP | ACCCTCGTTTTGCCATGAAC | TGTGCTGGGATTGATATTCCGTA |
| m-GAPDH | ACCACAGTCCATGCCATCAC | TCCACCACCCTGTTGCTGTA |
| m-β-catenin | CACGTGGGCTCCAGCATT | TCACCAGTCATTTCTGCCTTTG |
| m-P16 | GAACTCTTTCGGTCGTACCC | CGAATCTGCACCGTAGTTGA |
| m-P21 | CGAGAACGGTGGAACTTTGAC | CAGGGCTCAGGTAGACCTTG |
| m-IL1A | ATCAGTACCTCACGGCTGCT | TGGGTATCTCAGGCATCTCC |
| m-IL1B | GGGCCTCAAAGGAAAGAATC | CTCTGCTTGTGAGGTGCTGA |
| m-IL6 | TTCCATCCAGTTGCCTTCTT | CAGAATTGCCATTGCACAAC |
| m-IL8 | GTGCAGTTTTGCCAAGGAGT | CTCTGCACCCAGTTTTCCTT |
| m-MMP3 | ACACCGGATTTGCCAAGACA | CAGGCCCATCAAAAGGGACA |
| m-CCL2 | AGGTCCCTGTCATGCTTCTG | TCATTGGGATCATCTTGCTG |
| m-CCL5 | CTCACCATATGGCTCGGACA | TTCTTCTCTGGGTTGGCACA |
| m-CCL20 | CGACTGTTGCCTCTCGTACA | GCTTCATCGGCCATCTGTCT |
| m-CXCL1 | ACTCAAGAATGGTCGCGAGG | GTGCCATCAGAGCAGTCTGT |
| m-CXCL9 | CCTAGTGATAAGGAATGCACGATG | CTAGGCAGGTTTGATCTCCGTTC |
| m-CXCL10 | ATCATCCCTGCGAGCCTATCCT | GACCTTTTTTGGCTAAACGCTTTC |

m: mouse.

**Table S2: the antibodies used in our Western blot analysis.**

| anti-GAPDH | 1:5000 | Abclonal (AC002) |
| --- | --- | --- |
| anti-OCN | 1:1000 | Abclonal (A11530) |
| anti-β-TUBLIN | 1:5000 | Abclonal (AC021) |
| anti-METTL13 | 1: 1,000 | ABCAM (ab186002) |
| anti-RUNX2 | 1: 1,000 | Cell Signal Technology (8486) |
| anti-PPAR-γ | 1:1000 | Proteintech (16643-1-AP) |
| anti-Foxa1 | 1:1000 | GeneTex (GTX100308) |
| anti-HES-1 | 1:1000 | Abclonal (A0925) |
| anti-ALP | 1:1000 | SAB (#48608) |
| anti-FABP4 | 1:1000 | SANTA (#B1012) |
| anti-P53 | 3:1000 | Cell Signaling Technology (2527S) |
| anti-P21 | 1:500 | Abclonal (A1483) |
| anti-P16 | 1:1000 | Proteintech (10883-1-AP) |

**Table S3: the sequences of siRNAs.**

| **si-RNA** | **sense (5`-3`)** | **antisense (5`-3`)** |
| --- | --- | --- |
| METTL13 | GCAUCCAGAACAGAAACUUTT | AAGUUUCUGUUCUGGAUGCTT |
| Foxa1 | CAUGUCUAUGGACUUAAUATT | UAUUAAGUCCAUAGACAUGTT |
| HES-1 | GAUGCACUUAAGAAAGAUATT | UAUCUUUCUUAAGUGCAUCTT |
